# Macrophage MSR1 promotes BMSC osteogenic differentiation and M2-like polarization by activating PI3K/AKT/GSK3β/β-catenin pathway

**DOI:** 10.7150/thno.36930

**Published:** 2020-01-01

**Authors:** Shu-Jie Zhao, Fan-Qi Kong, Jian Jie, Qing Li, Hao Liu, An-Di Xu, Ya-Qing Yang, Bin Jiang, Dong-Dong Wang, Zhong-Qiu Zhou, Peng-Yu Tang, Jian Chen, Qian Wang, Zheng Zhou, Qi Chen, Guo-Yong Yin, Han-Wen Zhang, Jin Fan

**Affiliations:** 1 Department of Orthopedics, The First Affiliated Hospital of Nanjing Medical University, Nanjing, 210029, China.; 2 Department of Orthopedics, Pukou Branch of JiangSu Province Hospital (Nanjing Pukou Central Hospital), Nanjing, 211800, China.; 3 State Key Laboratory of Oncogenes and Related Genes, Shanghai Cancer Institute, Ren Ji Hospital, School of Medicine, Shanghai Jiao Tong University, Shanghai, 200240, China.; 4 Key Laboratory of Targeted Intervention of Cardiovascular Disease, Collaborative Innovation Center for Cardiovascular Disease Translational Medicine, Nanjing Medical University, Nanjing, 211100, China.; These authors contributed equally: Shu-Jie Zhao, Fan-Qi Kong and Jian Jie.

**Keywords:** Macrophage scavenger receptor 1, Bone marrow stem cells, Osteogenic differentiation, Oxidative phosphorylation, PI3K/AKT/GSK3β/β-catenin pathway

## Abstract

Approximately 10% of bone fractures do not heal satisfactorily, leading to significant clinical and socioeconomic implications. Recently, the role of macrophages in regulating bone marrow stem cell (BMSC) differentiation through the osteogenic pathway during fracture healing has attracted much attention.

**Methods**: The tibial monocortical defect model was employed to determine the critical role of macrophage scavenger receptor 1 (MSR1) during intramembranous ossification (IO) *in vivo*. The potential functions and mechanisms of MSR1 were explored in a co-culture system of bone marrow-derived macrophages (BMDMs), RAW264.7 cells, and BMSCs using qPCR, Western blotting, immunofluorescence, and RNA sequencing.

**Results**: In this study, using the tibial monocortical defect model, we observed delayed IO in MSR1 knockout (KO) mice compared to MSR1 wild-type (WT) mice. Furthermore, macrophage MSR1 mediated PI3K/AKT/GSK3β/β-catenin signaling increased ability to promote osteogenic differentiation of BMSCs in the co-culture system. We also identified proliferator-activated receptor gamma coactivator 1-alpha (PGC1α) as the target gene for macrophage MSR1-activated PI3K/AKT/GSK3β/β-catenin pathway in the co-culture system that facilitated M2-like polarization by enhancing mitochondrial oxidative phosphorylation.

**Conclusion**: Our findings revealed a previously unrecognized function of MSR1 in macrophages during fracture repair. Targeting MSR1 might, therefore, be a new therapeutic strategy for fracture repair.

## Introduction

Bone fracture is a severe global public health problem leading to an enormous burden of disability and suffering 1. Fracture repair is a highly complex but organized process, and proper healing requires the restoration of the damaged skeleton to its pre-injury cellular structure and biomechanical function 2, 3. Intramembranous and endochondral ossification are the two major pathways for bone regeneration 4, 5. During intramembranous ossification (IO), mesenchymal stem cells (MSCs) directly differentiate into osteoblasts and then deposit mineralized extracellular matrix 4, 6. Despite the available best treatment, nearly 10% of fracture patients still have poor outcomes 7. Recently, macrophages have been found to play an essential role during fracture healing through the release of paracrine cytokines regulating the recruitment and osteogenic differentiation of MSCs 8-11. Consequently, a better understanding of the cross-talk between macrophages and MSCs is expected to have a positive impact on fracture healing interventions.

In response to environmental signals, macrophages acquire different activation phenotypes, broadly classified into M1-like and M2-like polarization 12, 13. Notably, pathways are involved in regulating the macrophage phenotype and function 14, 15. Enhanced mitochondrial oxidative phosphorylation (OXPHOS) has been linked to the M2-like polarization phenotype 16, 17. The phosphatidylinositol 3-kinase/protein kinase B (PI3K/AKT) pathway not only affects the survival and migration but also orchestrates metabolic progress in macrophages 18. Glycogen Synthase Kinase 3 Beta (GSK3β), an important PI3K/AKT signaling substrate, displays a regulatory function on mitochondrial activities 19. Inhibition of GSK3β via its phosphorylation at Ser9 by activated PI3K/AKT signaling can lead to β-catenin stabilization and its translocation into the nucleus for gene transcription 19, 20. However, the mechanisms underlying the regulation of macrophage functions or metabolic characteristics by PI3K/AKT/GSK3β/β-catenin signaling remain elusive.

Macrophage scavenger receptor 1 (MSR1), also called scavenger receptor class A (SR-A) or CD204, was the first of the scavenger receptors to be characterized 21. MSR1 is mainly expressed in macrophages and known for its ability to scavenge modified lipoproteins 21. Recent evidence further points out that MSR1 participates in many pathophysiological events such as inflammation, virus recognition, and atherosclerosis 21, 22. MSR1 binding to various ligands has been shown to activate different signaling pathways such as PI3K/AKT, nuclear factor kappa B (NF-κB), and mitogen-activated protein kinase 21. However, the role of MSR1 in fracture healing remains unclear.

In the present study, delayed intramembranous bone healing was observed in MSR1 knockout (KO) mice compared with MSR1 wild-type (WT) mice in a tibial monocortical defect model. MSR1 in macrophages also regulated the osteogenic differentiation of bone marrow stem cells (BMSCs). We also demonstrated that loss of MSR1 in macrophages after co-culturing with BMSCs impaired its OXPHOS and M2-like polarization. Furthermore, our results revealed that MSR1 depletion in macrophages inactivated the PI3K/AKT/GSK3β/β-catenin signaling pathway in the co-culture system. Macrophage MSR1-activated PI3K/AKT/GSK3β/β-catenin signaling is essential for the increased osteogenic differentiation of BMSCs in a co-culture system. Notably, peroxisome proliferator-activated receptor gamma coactivator 1-alpha (PGC1α) was a critical target gene for MSR1-mediated PI3K/AKT/GSK3β/β-catenin signaling to facilitate M2-like macrophage activation by enhancing mitochondrial biogenesis.

## Materials and Methods

### Cell culture and reagents

The RAW264.7 cell line was obtained from the Cell Bank of the Chinese Academy of Sciences (Shanghai, China) and passed the test of DNA profiling (short tandem repeat (STR) profiling method). Primary bone marrow-derived macrophages (BMDMs) and BMSCs were obtained and cultured as previously described 22, 23. Flow cytometry (FACS Verse 8, BD, New York, USA) was used to identify the phenotypic surface biomarkers (positive biomarkers: CD90, CD73, and CD105; negative biomarkers: CD45 and CD34) of BMSCs according to the manufacturer's protocols. A tri-lineage-induced differentiation experiment (Osteogenic, chondrogenic, and adipogenic differentiation) was performed according to the manufacturer's instructions. Briefly, BMSCs were incubated in a 6-well plate at a density of 1 × 10^5^ cells/well in Mouse MSC Osteogenic (MUBMX-90021) or Adipogenic Differentiation Medium (MUBMX-90031) (Cyagen Biosciences, Sunnyvale, CA) for osteogenesis or adipogenesis induction, respectively. After 14 days, the cells were tested for adipogenesis using oil red O staining or osteogenesis using alizarin red (AR) staining. For chondrogenesis, pellet culture was used. 1 × 10^6^ cells/tube was cultured in Mouse MSC chondrogenic Differentiation Medium (MUBMX-9004, Cyagen Biosciences) for chondrogenesis induction. After 21 days, the pellet was fixed embedded and analyzed for chondrogenesis by Alcian Blue staining. To test osteogenic differentiation of BMSCs in the co-culture system, BMSCs were plated with macrophages (BMDMs or RAW264.7 cells) in a Transwell chamber (0.4 μm pore) at 1:10 ratio. BMSCs were cultured in the MUBMX-90021medium at the bottom of the well for 1-2 weeks, and the medium was changed twice a week. To investigate the polarization phenotype of macrophages, BMSCs were cultured in the upper chamber, and macrophages were seeded in the lower chamber. mRNA expression levels of M1-like biomarkers (iNOS and IL1β) and M2-like biomarkers (CD206 and CD163) were determined using qPCR. Additionally, to evaluate the migration of BMSCs, non-activated or LPS-activated (L2880, Sigma-Aldrich, MO, USA) BMDMs (from MSR1 KO and WT mice) were seeded with BMSCs in another Transwell system (8.0 μm pore). After 24 h, the migrated BMSCs were fixed and stained with 0.1% crystal violet.

The antibodies for Western blotting in our study included anti-β-actin (AB0011, Abways, Shanghai, China), anti-Histone H3 (3638, CST, MA, USA), anti-MSR1 (ab123946, Abcam, Cambridge, UK), anti-AKT (4691, CST), anti-pAKT (4060, CST), anti-GSK3β (12456, CST), anti-pGSK3β (5558T, CST), anti-mTOR (2972, CST), anti-p-mTOR (5536, CST), and anti-β-catenin (8480, CST). Secondary antibodies for Western blotting were purchased from Jackson ImmunoResearch (West Grove, PA, USA). The antibodies for IF were anti-iNOS (ab15323, Abcam), anti-CD206 (ab64693, Abcam), anti-F4/80 (14-4801-82, Thermo, Massachusetts, USA), anti-β-catenin (8480, CST) and secondary antibodies for IF were donkey anti-mouse Alexa Fluor 488 (ab150105, Abcam), goat anti-rabbit Alexa Fluor 594 (ab150088, Abcam) and goat anti-rabbit Alexa Fluor 647 (ab150083, Abcam). The antibodies for flow cytometry consisted of F4/80-PE (565410, BD, New York, USA), iNOS-FITC (610330, BD), CD206-APC (17-2061-82, Thermo), CD90-FITC (ab25672, Abcam), CD73-PE (550741, BD), CD105-FITC (ab184667, Abcam), CD45-PE (561087, BD), and CD34-FITC (553733, BD). To inhibit PI3K or AKT, 10 μM LY294002 (S1105, Select, Houston, USA) or 1 μM ARQ 092 (S8339, Select) was used, respectively. The BMP4 ELISA kit was obtained from CUSABIO (CSB-E04512m, WuHan, China).

### Tibial monocortical defect model

MSR1 KO mice (C57BL/6 background) were acquired as previously described, and WT mice with identical genetic backgrounds were used as controls 24. Genotyping was performed by PCR analysis of DNA samples that were isolated from tail chips. All mice were housed and handled in compliance with the Animal Committee at the First Affiliated Hospital of Nanjing Medical University. For the tibial monocortical defect model, a monocortical osseous hole (0.8 mm diameter) was created on the anterior surface of the tibia crest using a round burr attached to a dental drill (NSK Ultimate XL, Japan) after administering adequate anesthesia as previously described 25, 26. Subsequently, the soft-tissue wound was closed, and buprenorphine was used as an analgesic.

### Micro-computed tomography (micro-CT) imaging

For the tibial monocortical defect model, the tibias were obtained on day 7 and 14 post-surgery. All tissues were fixed overnight with 4% paraformaldehyde. The tibias were scanned and reconstructed with 18 μm resolution using micro-CT analysis system (SkyScan 1176, Bruker, Germany). The reconstruction of three-dimensional images and the analysis of morphometric parameters were performed using CT-Analyzer (CTAn, Bruker, Germany). For the post-surgery tibias, the lower grey level was defined at 60, and the upper grey level was defined at 255.

### Tartrate-resistant acid phosphatase (TRAP) staining

TRAP staining was performed as previously described 27. Briefly, decalcified sections from tibial monocortical defect model were incubated for 45-60 min at 37 °C using TRAP staining solution, according to the manufacturer's protocol (387A-1KT, Sigma). Next, the slides were counterstained with hematoxylin solution and were photographed.

### Immunofluorescence (IF) assay

The IF assay was performed as previously reported [2d7-29]. Cells were fixed with 4% paraformaldehyde and then permeabilized with 0.05% Triton X-100 for 1-2 min. Next, cells were blocked with 5% BSA for 1 h and incubated with the following specific antibodies overnight at 4 °C: anti-F4/80 (1:200), anti-iNOS (1:200), and anti-CD206 (1:200). Subsequently, the species-matched secondary antibodies were used, and the nucleus was stained with DAPI. For tissue IF staining, the slides were deparaffinized, hydrated, and were subjected to heat-mediated antigen retrieval in sodium citrate antigen retrieval solution (C1032, Solarbio, Beijing, China). After blocking with 10% BSA, anti-F4/80 (1:100), anti-iNOS (1:100), and anti-CD206 (1:100) antibodies were used. The secondary antibodies were donkey anti-mouse Alexa Fluor 488 (1:400), goat anti-rabbit Alexa Fluor 594 (1:400,) and goat anti-rabbit Alexa Fluor 647 (1:300). The nuclei were stained DAPI, and images were acquired with a confocal microscope (Zeiss LSM710, Heidenheim, Germany).

### Plasmid construction and transfection

The plasmid containing full-length MSR1 and a negative control plasmid were purchased from GenePharma (Shanghai, China). Virus packaging was performed as previously described 27-29, and titers were also tested. The cells were infected with 1 × 10^8^ lentivirus-transducing units in the presence of 5 μg/mL polybrene (GenePharma, Shanghai, China). After 72 h of culture, infected cells were further selected with 2.5 μg/mL puromycin. Overexpression efficacy of MSR1 was verified by qPCR and Western blotting.

### RNA isolation and qPCR

Total RNA of cells and callus was isolated using the Trizol reagent (Takara, Dalian, China) following the manufacturer's instruction, and transcribed into cDNA using the HiScript II Q RT SuperMix for qPCR (R122-01, Vazyme, China). Next, qPCR was performed using AceQ qPCR SYBR Green Master Mix (Q111-02, Vazyme, China) in a 7500 real-time PCR system (Applied Biosystems, Inc., USA). The primer sequences are listed in Table S1. All data were normalized to β-actin expression. Quantification of qPCR results was performed by the 2^-△CT^ method.

### RNA sequence (RNA-seq) and gene set enrichment analysis (GSEA)

Total RNA of macrophages from MSR1 KO and WT groups (n = 3) was extracted. Next, quality RNA samples were converted into cDNA libraries using VAHTSTM mRNA-seq V2 Library Prep Kit from Illumina® (NR601, Vazyme, Nanjing, China). VAHTSTM DNA Clean Beads (Vazyme #N411) were used to purify the fragments during the process of library generation. The purified products were enriched with 12-15 cycles of PCR to create the final cDNA library. Finally, libraries were sequenced on the Illumina Hiseq X Ten according to the manufacturer's protocols. The reads were aligned with TopHat program (version 2.0.11). Additionally, the FPKM values of genes were calculated, Pearson's correlation analysis was performed, and heatmaps were generated. The results of RNA-seq were uploaded to the Gene Expression Omnibus (GEO) database under accession number GSE134693. In our study, differentially expressed genes (DEGs) were defined as fold changes > 1.5 and P < 0.05. The Kyoto Encyclopedia of Genes and Genomes (KEGG) analysis and GSEA were further performed to interpret the biological significance of DEGs.

### Western blotting

Western blotting was carried out as previously described 27-29. The protein extraction buffer (Beyotime, Shanghai, China) or nucleoprotein extraction kit (Sangon Biotech, C500009) was used to extract total or nuclear cellular proteins according to the manufacturer's instructions. Further, equal amounts of proteins were separated by SDS-PAGE gel electrophoresis and transferred onto a polyvinylidene fluoride (PVDF) membrane. After blocking with 5% skimmed milk or 5% Bovine Serum Albumin, the membrane was probed with the following primary antibodies: anti-β-actin (1:2000), anti-Histone H3 (1:1000), anti-MSR1 (1:1000), anti-AKT (1:1000), anti-p-AKT (1:1000), anti-GSK3β (1:1000), anti-p-GSK3β (1:1000), anti-mTOR (1:1000), anti-p-mTOR (1:1000), and anti-β-catenin (1:1000). The species-matched secondary antibodies were used (1:10000), and the bands were detected by the Odyssey imaging system (LI-COR, Lincoln, NE, USA).

### Alizarin red staining and alkaline phosphatase (ALP) enzyme assay

Co-cultured BMSCs were first fixed with 4% paraformaldehyde for 30-45 min, then stained with 2% alizarin red to detect the extent of matrix mineralization. Alizarin red was further isolated with cetylpyridinium chloride and was detected at an absorbance value of 562 nm using a spectrophotometer. To evaluate the deposited mineral, the activity of ALP was analyzed with Alkaline Phosphatase Assay Kit according to the manufacturer's instructions (P0321, Beyotime, ShangHai, China).

### Flow cytometry

Co-cultured BMDMs or RAW 264.7 cells were collected and stained with F4/80-PE, CD11C-PE-CyTM7, and CD206-APC according to the manufacturer's instructions. BMSCs were obtained and incubated with CD90-FITC, CD73-PE, CD105-FITC, CD45-PE, and CD34-FITC. Subsequently, the cells were analyzed by flow cytometry (FACSVerse 8, BD), and data analysis was performed using the FlowJo software (Version 7.6.1, Treestar, USA).

### Enzyme-linked immunosorbent assay (ELISA)

ELISA was employed to assess the secretion of BMP4 by macrophages following the manufacturer's instructions. The absorbance was determined using a microplate reader (BioTek, Friedrichshall, Germany) at 450 nm.

### Measurement of OXPHOS

The mitochondrial OXPHOS of macrophages in a co-culture system was measured using the XF96 metabolic flux analyzer (Seahorse Biosciences, Billerica, MA, USA) as previously described 28. The O2 consumption rate (OCR) was tested by the sequential addition of 2 μM oligomycin, 1 μM carbonyl cyanide 4-(trifluoromethoxy) phenylhydrazone (FCCP) (C2920; Sigma-Aldrich, C2920), 1 μM antimycin A, and 1 μM rotenone (A&R) (Sigma-Aldrich). The results were quantified by the XFe Wave software (Seahorse Biosciences, California, USA). Upon completion of real-time OCR measurement after sequential injection of the indicated inhibitors, the modulating mitochondrial function, basal respiration, ATP production, respiratory capacity (maximal electron transport chain activity), and respiratory reserve (flexibility with increased energy demand) were calculated following the manufacturer's protocol.

### Transmission electron microscopy (TEM)

The cells were collected and fixed with 2.5% glutaraldehyde overnight at 4 °C as previously reported 27. The samples were fixed, dehydrated, stained, embedded, sectioned at 70 nm, and visualized using a transmission electron microscope (Tecnai G2 Spirit Bio TWIN, FEI, USA).

### Chromatin immunoprecipitation (ChIP) assay

Primary macrophages were first fixed using 1% (w/v) formaldehyde for 10 min at 37°C. Based on previous reports, the following steps were performed using the Pierce Agarose ChIP kit (26156, Thermo) 28, 29. After crosslinking and sonication, the DNA was immunoprecipitated with corresponding antibodies (Transcription Factor 4 (TCF4)-specific antibody or rabbit IgG) overnight at 4°C. Next, the DNA was analyzed by PCR, using SYBR® Green master mix and primers (Takara, Japan). The primers used for the ChIP assay are listed in Table S1.

### Luciferase reporter assay

To confirm the binding sites of TCF4 and PGC1α, luciferase reporter plasmids containing wild-type and mutant PGC1α promoters were inserted into the PGL3B vector (Table S1). Macrophages were co-transfected with TCF4 and PGL3B-PGC1α vectors using Lipofectamine 3000 (Invitrogen, Carlsbad, CA, USA). Following 48 h of transfection, cells were obtained, and luciferase activity was quantified with the Luciferase Reporter Assay System (Promega, Madison, WI, USA). Renilla luciferase activity was used to normalize for transfection efficiency.

### Bone marrow transplantation

As previously reported, the recipient mice (8 weeks) were irradiated at 700 cGray using an X-ray orthovoltage source (RS 2000 Pro, RADSOURCE, USA) before transplantation 24. A total of 5 × 10^6^ marrow cells were harvested from donor mice (MSR1 WT or KO mice) and introduced via tail vein injection into lethally irradiated recipients. Two weeks before and after transplantation, the recipient mice were provided water containing neomycin and polymyxin B. Four weeks after transplantation, tibial monocortical defect surgery was performed on the recipient mice.

### Statistical analyses

Data are presented as mean ± SD and contain at least three independent biological replicates. One-way analysis of variance was performed if comparisons were more than two groups, and unpaired two-tailed student's t-test was used for two-group comparisons with GraphPad Prism 7 (GraphPad Software, La Jolla, CA, USA). Differences between groups were considered significant at a p-value < 0.05.

## Results

### MSR1 deficiency impairs intramembranous ossification

**Figure 1:**
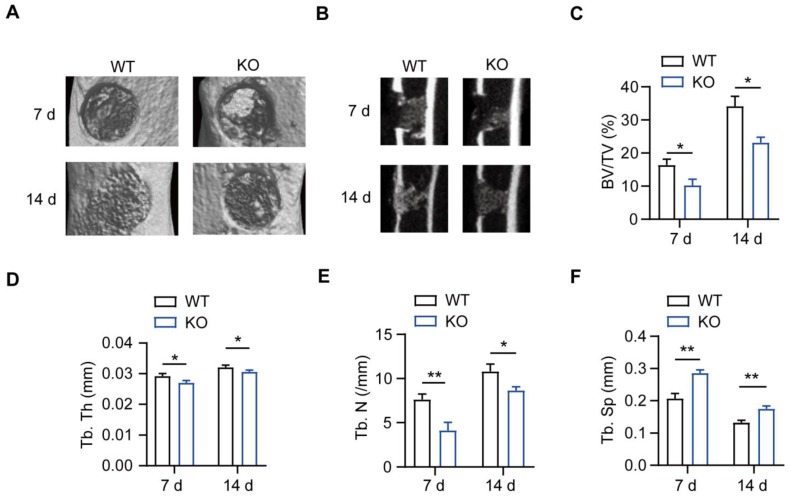
** Impaired intramembranous ossification (IO) in MSR1 KO mice.** (A) Representative 3D images of the injured tibiae by micro-CT on day 7 or 14 post-surgery. (B) Representative 2D coronal images of the injured tibiae from MSR1 WT and KO groups on day 7 or 14 post-surgery. (C-F) 3D structural parameters of bone volume (BV)/tissue volume (TV) (%) (C), trabecular thickness (Tb. Th) (D), trabecular number (Tb. N) (E) and trabecular separation (Tb. Sp) (F) for the defect region on day 7 and 14 post-surgery were further analyzed (values are mean ± SD, *p < 0.05, **p < 0.01).

MSR1 WT and MSR1 KO mice were used to explore the role of MSR1 during IO, and genotyping was confirmed via PCR of DNA samples from tail chips (Figure S1A). A simplified stable fracture model with the tibial monocortical defect was employed to explore the role of MSR1 in IO. Compared to MSR1 WT mice, delayed intramembranous bone repair was observed in MSR1 KO mice using micro-CT (Figures 1A and B). Decreased bone volume/tissue volume (BV/TV), trabecular thickness (Tb. Th), trabecular number (Tb. N) and increased trabecular separation (Tb. Sp) were detected on day 7 and 14 post-surgery in MSR1 KO mice compared to the MSR1 WT mice (Figures 1C-F). Collectively, these results suggested that MSR1-knockout might lead to impaired IO *in vivo*.

### Macrophage MSR1 promotes BMSC osteogenic differentiation *in vitro*

**Figure 2:**
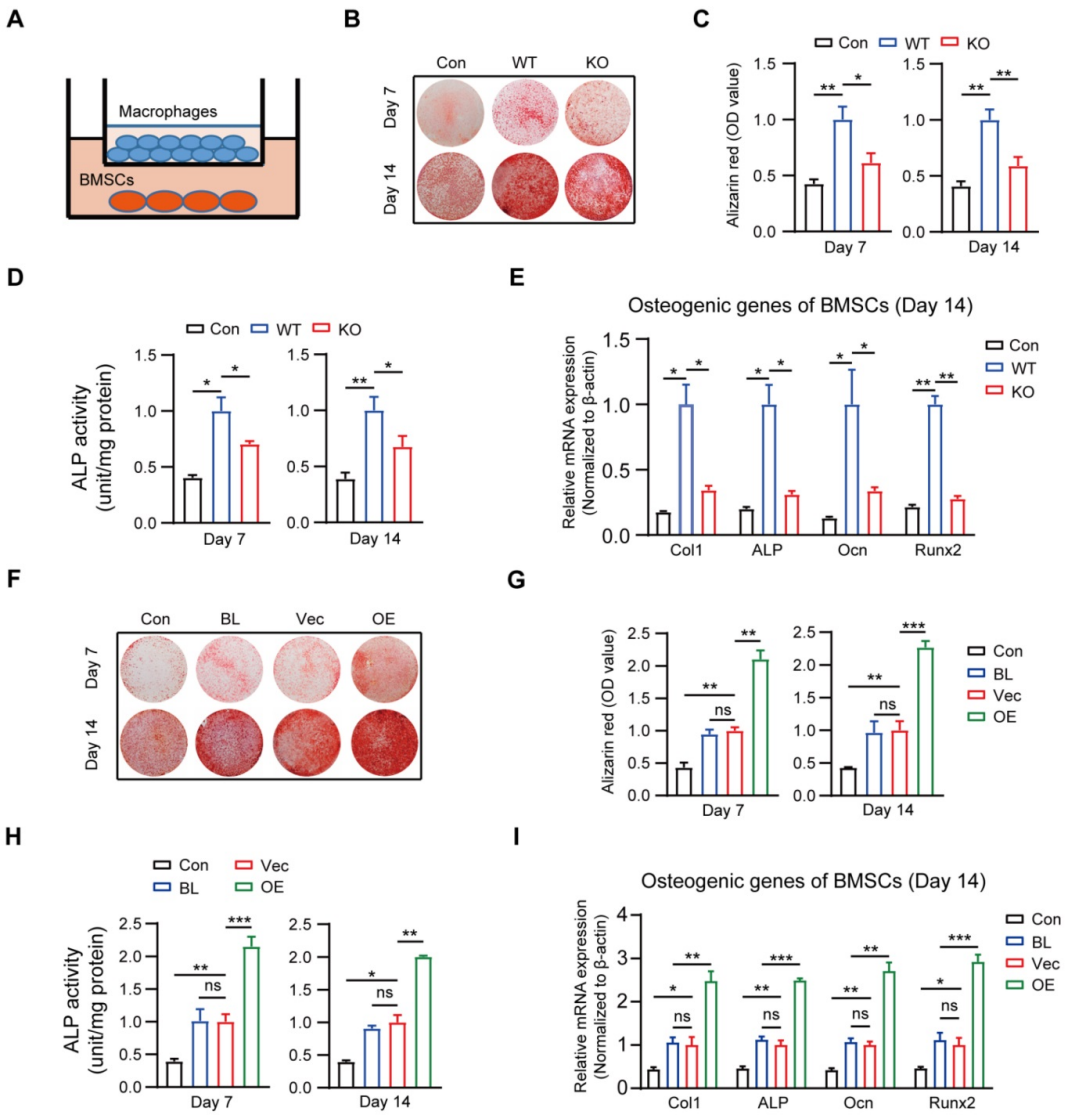
** Macrophage MSR1 exhibits pro-osteogenic differentiation effect of BMSC in a co-culture system.** (A) BMSCs were seeded in the lower chamber, and macrophages were cultured in the upper chamber. (B) In a co-culture system for 7 or 14 days, MSR1 KO BMDMs reduced the ability to promote osteogenic differentiation of BMSCs as indicated by AR staining. BMSCs without co-culture were set as the control (Con) group. (C and D) Quantitative evaluation of AR staining results (C) and ALP activities (D) on day 7 and 14 was performed (Values are expressed as mean ± SD, *p < 0.05, **p < 0.01). (E) mRNA expression levels of osteogenic marker genes (Runx2, Ocn, ALP, and Col1) in osteogenic differentiation of BMSCs on day 14 were detected by qPCR in different groups. β-actin was used as an internal control (Values are mean ± SD, *p < 0.05, **p < 0.01). (F) In the co-culture system, MSR1-overexpressing RAW264.7 cells enhanced osteogenic differentiation of BMSCs on day 7 and 14 as revealed by AR staining. BMSCs cultured alone were set as the Con group and RAW264.7 cells without MSR1-plasmid transfection were defined as the blank (BL) group. Vec: vector group, OE: overexpression group. (G and H) Quantitative analyses of AR staining results (G) and ALP activities (H) of osteogenic differentiation of BMSCs on day 7 and 14 were performed. Values are expressed as mean ± SD, *p < 0.05, **p < 0.01, ***p < 0.001, ns indicates no significance. (I) mRNA expression levels of Col1, ALP, Ocn and Runx2 in osteogenic differentiation of BMSCs on day 14 by qPCR in different groups. β-actin was used as an internal control (Values are expressed as mean ± SD, *p < 0.05, **p < 0.01, ***p < 0.001, ns indicates no significance).

It is generally accepted that IO depends on the recruitment and subsequent differentiation of MSCs, and chemokines and cytokines derived from macrophages contribute to the migration and differentiation of MSCs for bone regeneration 30-32. Because MSR1 has been reported to be primarily expressed on macrophages, we investigated whether MSR1 participated in modulating BMSC migration and/or osteogenic differentiation *in vitro*. BMSCs were obtained and verified for plastic-adherence and negative expression of surface biomarkers CD45 and CD34, but a positive expression of CD105, CD73, and CD90 as well as tri-lineage differentiation (osteogenic, chondrogenic, and adipogenic differentiation) ability *in vitro* (Figures S2A and B). BMDMs were also isolated from MSR1 WT mice, and Western blotting revealed that MSR1 was expressed only on BMDMs (Figure S2C). The knockout efficiency of MSR1 on BMDMs was tested through qPCR and Western blotting (Figure S2D). As shown in Figure S2E, a co-culture system (8.0 μm pore) was used in which non-activated or LPS-activated BMDMs from MSR1 WT or KO mice were seeded in the lower chamber and BMSCs were cultured in the upper chamber. Compared to the MSR1 WT group, the MSR1 KO macrophages did not alter the migration ability of BMSCs (Figures S2F and G). Furthermore, in another co-culture system (0.4 μm pore), the results of AR staining and subsequent quantitative evaluation revealed that MSR1-depleted BMDMs partially impaired the enhancement of osteogenic differentiation effect of BMSCs (Figures 2A-C). Statistical analysis of ALP activities and mRNA expression levels of osteogenic marker genes (Col1, ALP, Ocn and Runx2) also verified the above results (Figures 2D and E, and Figure S2H).

The results mentioned above suggested that macrophage MSR1 mainly contributed to the pro-osteogenic differentiation effect of BMSCs in the co-culture system. RAW264.7 cells were used to further reinforce this conclusion. As shown in Figure S2I, MSR1 was overexpressed on RAW264.7 cells which were confirmed by qPCR and Western blotting. As expected, AR staining showed significantly enhanced osteogenic differentiation of BMSCs, and higher ALP activity was found after co-culturing with MSR1-overexpressing RAW264.7 cells (Figures 2F-H). Also, mRNA expression values of Col1, ALP, Ocn and Runx2 were elevated in MSR1-overexpressing RAW264.7 cells on days 7 and 14 in the co-culture system (Figure 2I and Figure S2J). Collectively, these results indicated that macrophage MSR1 might lead to pro-osteogenic differentiation of BMSCs in the co-culture system.

### Role of MSR1 in the infiltrated macrophages during intramembranous ossification

**Figure 3:**
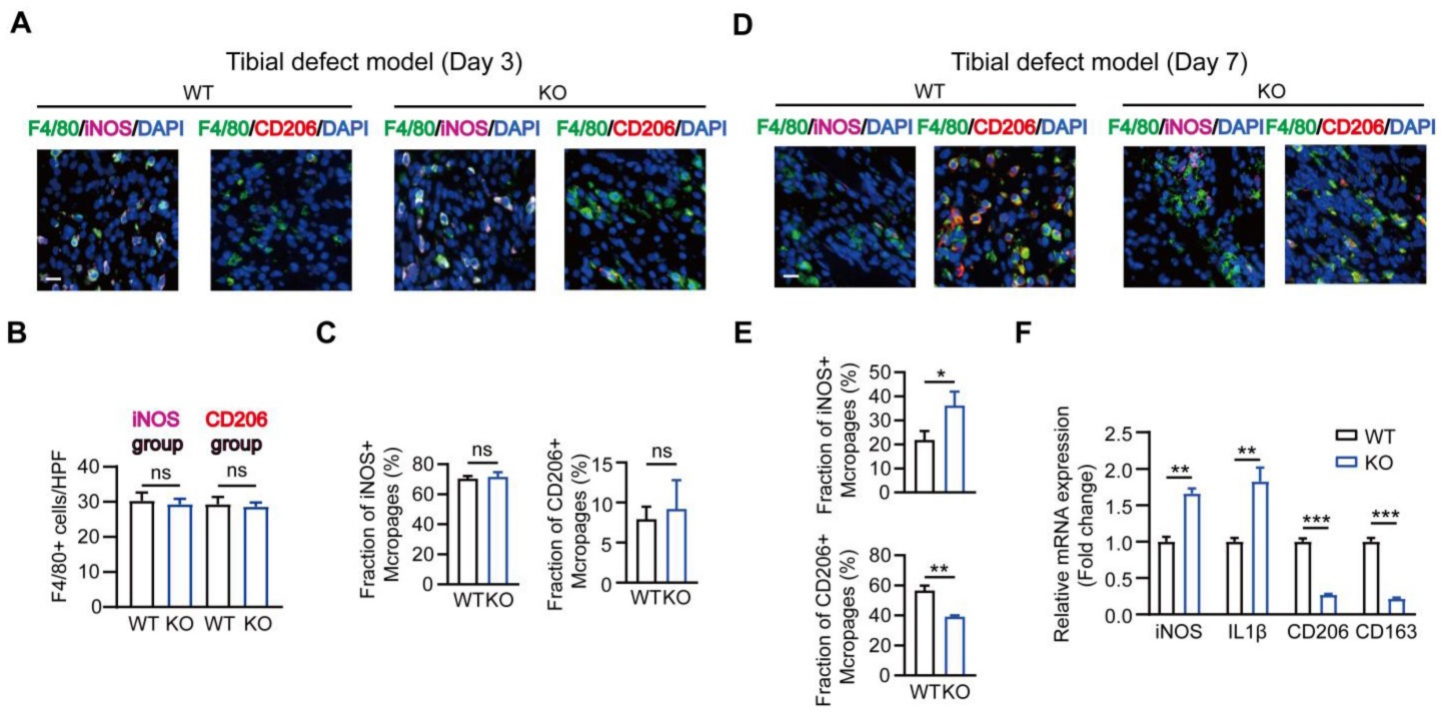
** MSR1-depletion reduced the fraction of M2-like macrophages on day 7 post-surgery in the tibial monocortical defect model.** (A) Immunofluorescence (IF) staining of the total macrophage biomarker, F4/80 (green), and M1-like macrophage biomarker, iNOS (purple) and M2-like macrophage biomarker, CD206 (red), in facture tissues on day 3 post-surgery in the tibial monocortical defect model. Nuclei were counterstained with DAPI (blue). Bar = 200 μm. (B and C) The infiltration of F4/80^+^ macrophages (B) and the fraction of iNOS^+^ F4/80^+^ and CD206^+^ F4/80^+^ macrophages (C) were determined on day 3 post-surgery in the tibial monocortical defect model from MSR1 WT and KO mice (Values are expressed as mean ± SD, ns indicates no significance). iNOS group indicates the samples stained with anti-F4/80 and anti-iNOS; the slides stained with anti-F4/80, and anti-CD206 denote the CD206 group. (D) Representative IF images of total macrophages (F4/80^+^), M1-like macrophages (iNOS^+^ F4/80^+^), and M2-like macrophages (CD206^+^ F4/80^+^) in facture tissues on day 7 post-surgery of the tibial monocortical defect model. Nuclei were counterstained with DAPI (blue). Bar = 200 μm. (E) The iNOS^+^ and CD206^+^ macrophage fractions were determined by the percentages of iNOS^+^ and CD206^+^ macrophages within F4/80^+^ macrophage populations in the MSR1 WT or KO fracture tissues on day 7 post-surgery in the tibial monocortical defect model (Values are expressed as mean ± SD, *p < 0.05, **p < 0.01). (F) mRNA expression levels of macrophage marker genes (M1-like: iNOS and IL-1b, M2-like: CD206 and CD163) in the fracture tissues from MSR1 WT or MSR1 KO mice on day 7 in the tibial monocortical defect model (L) (*p < 0.05, **p < 0.01, ***p < 0.001).

It is known that M1-like macrophages exhibit pro-inflammatory functions, while the M2-like type is characterized by the production of anti-inflammatory cytokines displaying potent tissue remodeling properties 12. Therefore, we explored the effect of MSR1 on macrophage phenotype polarization during intramembranous ossification. As shown in Figures 3A-C, in the tibial monocortical defect model, M1-like macrophages (F4/80^+^ and iNOS^+^) were the dominant population on day 3 post-surgery. However, there was no significant difference in the infiltration and polarization of macrophages between MSR1 KO and WT mice at this time point (Figures 3A-C). These results suggested that the acute and complex inflammatory microenvironment could facilitate M1-like macrophage polarization and MSR1 might not be involved in the early inflammatory response during fracture healing. From 3 to 7 days post-surgery, M1-like macrophages were gradually replaced by M2-like macrophages for tissue repair 33, 34. Furthermore, on day 7 post-surgery, we studied the polarization phenotype of macrophages in the fractured sites of the model. As indicated in Figures 3D and E, and Figure S3A, there was no significant difference in the infiltration of F4/80^+^ macrophages, but a significantly increased M1-like macrophage (F4/80^+^ and iNOS^+^) fraction and a markedly decreased M2-like macrophages (F4/80^+^ and CD206^+^) were observed in the MSR1 KO mice. The mRNA expression analysis of the injury sites further revealed that M1-like biomarkers (iNOS and IL1β) increased and M2-like biomarkers (CD206 and CD163) decreased in the MSR1 KO mice compared to MSR1 WT mice on day 7 post-surgery (Figure 3F). We also explored if MSR1 affected the number of osteoclasts (OCs) in the tibial monocortical defect model. The results of TRAP staining showed no significant difference in the number of OCs between MSR1 KO and WT mice on day 7 post-surgery in this model (Figure S3B).

Collectively, our results suggested that MSR1-depletion could reduce the infiltrated M2-like macrophages on day 7 post-surgery.

### Role of macrophage MSR1 in mitochondrial OXPHOS and M2-like polarization after co-culture with BMSCs

**Figure 4:**
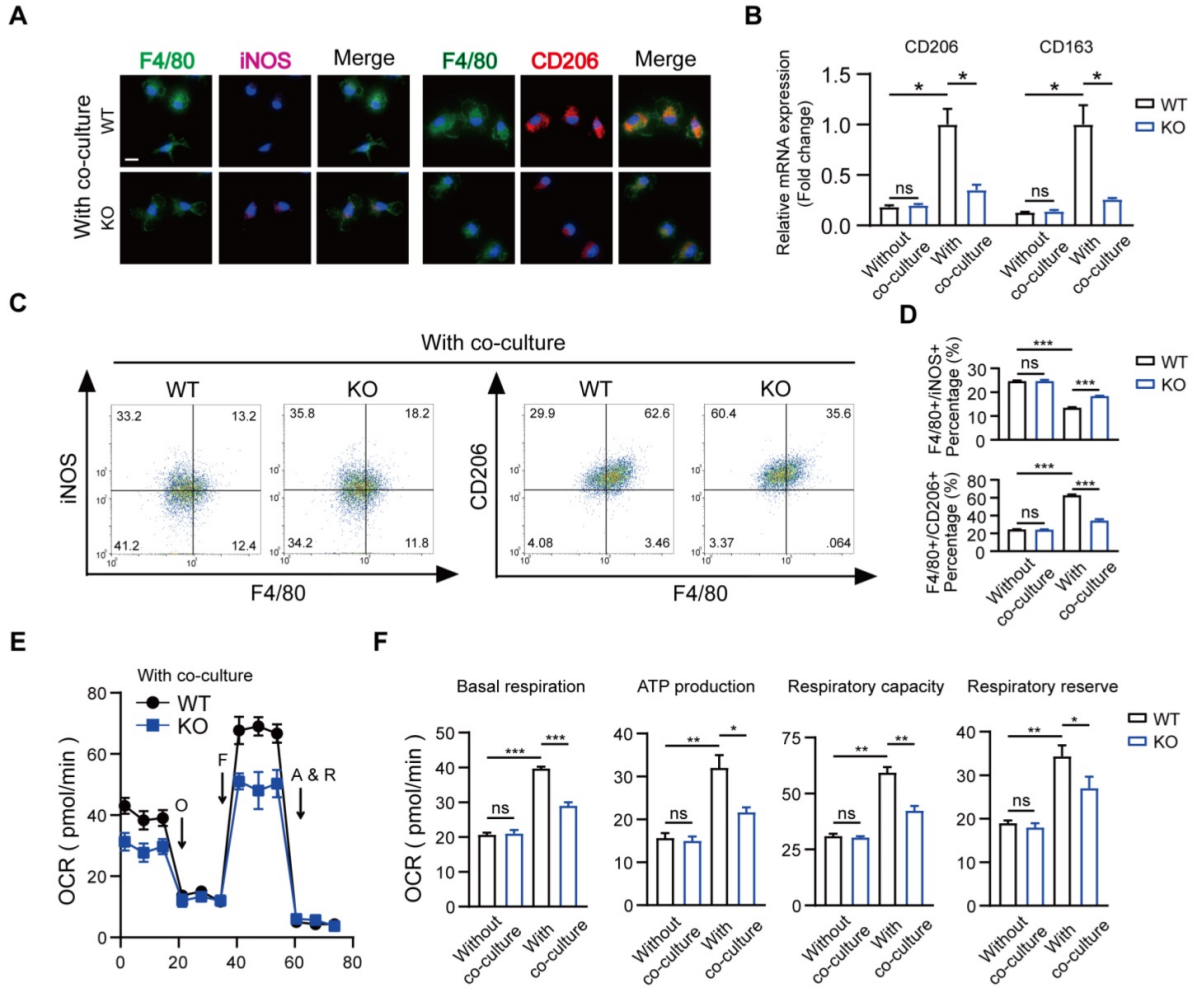
** Depletion of MSR1 in BMDMs reduced M2-like macrophages and mitochondrial OXPHOS in the co-culture system.** (A) IF staining results of MSR1 WT and KO macrophages in the co-culture system for an M1-like marker (iNOS) and M2-like marker (CD206). Bar = 50 μm. (B) mRNA expression levels of M2-like marker genes (CD206 and CD163) in MSR1 WT and KO macrophages with or without co-culture by qPCR. Values are mean ± SD, *p < 0.05, ns indicates no significance. (C) Flow cytometry analysis of MSR1 WT or KO macrophages after co-culturing with BMSCs. Dot plots represent F4/80 and iNOS staining (left panel) and F4/80 and CD206 staining of macrophages (right panel). (D) Percentages of F4/80^+^ iNOS^+^ and F4/80^+^ CD206^+^ macrophages with or without co-culture were determined. Values are mean ± SD, ***p < 0.001, ns indicates no significance. (E) OCR of BMDMs in MSR1 WT or KO group after co-culture was detected using a Seahorse Bioscience XFp analyzer. O: Oligomycin, F: FCCP, A&R: antimycin A/rotenone. (F) Mitochondrial activities such as basal respiration, ATP production, respiratory capacity, and respiratory reserve were determined in indicated groups. Values are mean ± SD, *p < 0.05, **p < 0.01, ***p < 0.001, ns indicates no significance.

Recently, immune-modulating characteristics of BMSCs in macrophages that contribute to tissue repair have been described, but the exact mechanisms remain to be determined 35, 36. We used a co-culture system and mainly focused on the role of MSR1 during tissue repair. The results of qPCR indicated that compared to BMDMs alone, those co-cultured with BMSCs for 2 days were remarkably polarized to the M2-like phenotype (Figures S4A and B). Also, a slightly stronger purple (iNOS) fluorescence intensity and much weaker red (CD206) fluorescence intensity were observed in MSR1 KO macrophages in the co-culture system (Figure 4A). The mRNA expression levels of M1-like biomarkers (iNOS and IL1β) significantly increased, and those of the M2-like biomarkers (CD206 and CD163) markedly decreased in the MSR1 KO macrophages compared to MSR1 WT macrophages when co-cultured with BMSCs (Figure 4B and Figure S4C). As displayed in Figures 4C and D, higher percentages of F4/80^+^ iNOS^+^ cells and lower percentages of F4/80^+^ CD206^+^ cells were detected in the MSR1 KO group. However, there was no significant difference in the polarized BMDMs between the MSR1 WT and KO groups without the co-culture (Figures 4B and D, and Figures S4C-E).

Numerous studies focused on the changes in macrophage mitochondrial OXPHOS, which may alter its polarization phenotype 37-39. To verify whether MSR1 modulated the changes in mitochondrial respiration, we performed a metabolic flux analysis after co-culturing macrophages with BMSCs. As displayed in Figures 4E and S4F, compared to BMDMs alone, there was a significant increase in the OCR, which is a biomarker of oxidative phosphorylation, upon co-culturing BMDMs with BMSCs. Also, the basal respiration, ATP production, respiratory capacity, and respiratory reserve of BMDMs were significantly increased after co-culturing with BMSCs (Figure 4F). To determine whether mitochondrial OXPHOS of BMDMs was important in M2-like activation in this co-culture system, 1 μM antimycin and rotenone (inhibitors of OXPHOS) were used. As shown in Figures S4G and S4H, the effect of BMSCs in modulating macrophage M2-like polarization in the co-culture system was partly decreased when OXPHOS was inhibited. Further, as shown in Figures 4E and F, there was a reduction in OCR as well as basal respiration, ATP production, respiratory capacity, and respiratory reserve following MSR1 knockout in BMDMs. However, there was no significant difference in OCR of BMDMs between MSR1 WT and KO groups without co-culturing (Figure 4F and Figure S4F).

**Figure 5:**
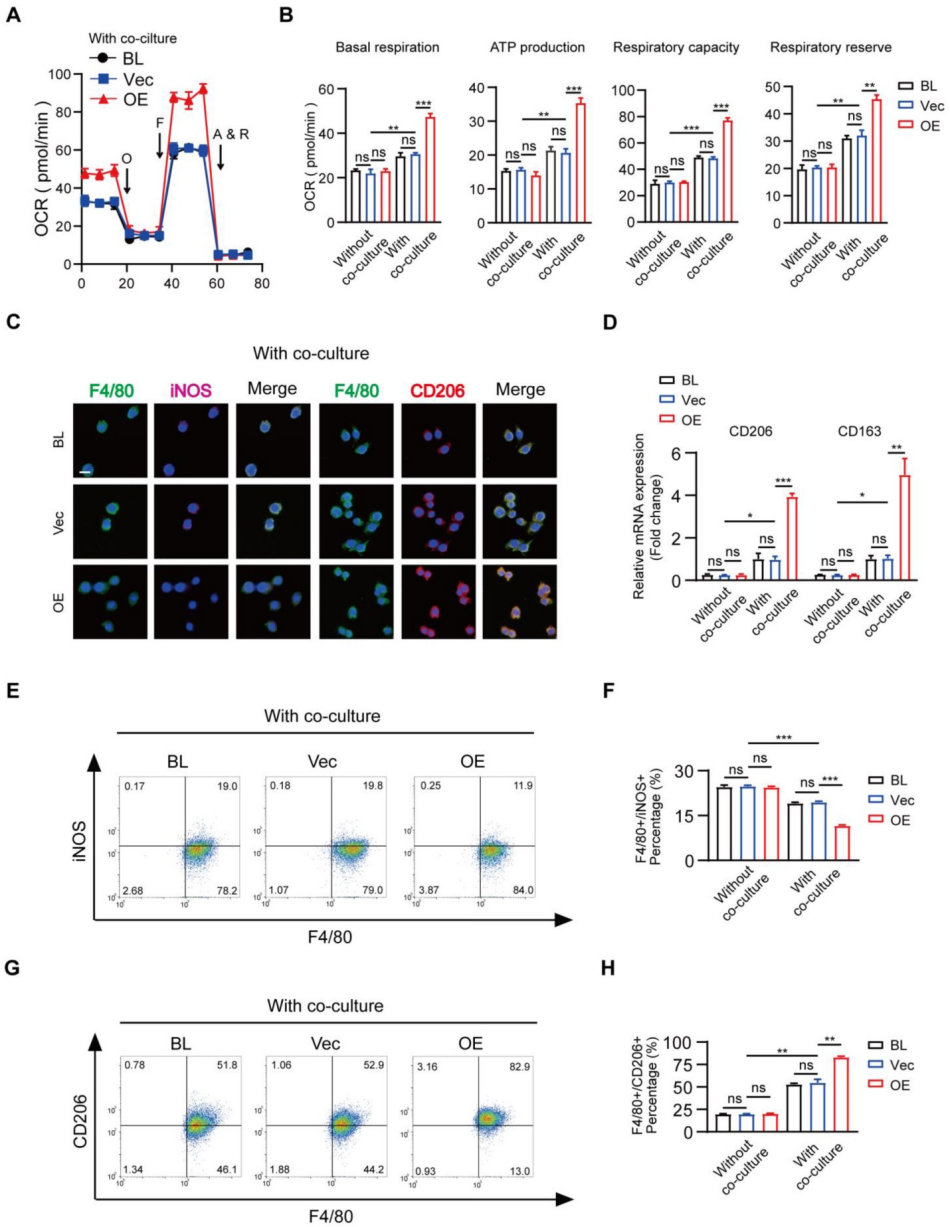
** Overexpression of MSR1 in RAW264.7 cells promotes mitochondrial OXPHOS and M2-like polarization in the co-culture system.** (A) OCR of blank (BL), vector (Vec), and MSR1-overexpressed (OE) RAW264.7 cells after co-culture were evaluated using a Seahorse Bioscience XFp analyzer. (B) Basal respiration, ATP production, respiratory capacity, and respiratory reserve were determined in BL, Vec, and MSR1 OE RAW264.7 cells with or without co-culture. Values are expressed as mean ± SD, **p < 0.01, ***p < 0.001, ns indicates no significance. (C) IF staining of BL, Vec, and OE RAW264.7 cells after co-culture for the M1-like biomarker (iNOS) and M2-like biomarker (CD206). Bar = 50 μm. (D) mRNA expression levels of M2-like macrophage marker genes (CD206 and CD163) in indicated groups were analyzed by qPCR. Values are expressed as mean ± SD, *p < 0.05, **p < 0.01, ***p < 0.001, ns indicates no significance. (E and G) Flow cytometry analysis of RAW264.7 cells from different groups after co-culture with BMSCs. Dot plots represent F4/80 and iNOS staining of (E) and F4/80 and CD206 staining of RAW264.7 cells (G). (F and H) percentages of F4/80^+^ iNOS^+^ (F) and F4/80^+^ CD206^+^ (H) in RAW264.7 cells in different groups were calculated. Values are expressed as mean ± SD, *p < 0.05, **p < 0.01, ***p < 0.001, ns indicates no significance.

Because of the previously mentioned functions of BMDM MSR1 in mitochondrial OXPHOS, overexpression of MSR1 in RAW264.7 cells induced a significantly up-regulated value of OCR in the co-culture system (Figure 5A and Figure S5A). Quantification of basal respiration, ATP production, respiratory capacity, and respiratory reserve showed an increase in MSR1-overexpressing RAW264.7 cells after co-culturing with BMSCs (Figure 5B). Furthermore, we analyzed the role of MSR1 overexpression in the polarization phenotype alteration for RAW264.7 cells in the co-culture system. As shown in Figures 5C-H and Figures S5B-D, the variation in the expression levels of M1-like or M2-like biomarkers was evaluated using fluorescence staining, qPCR, and flow cytometry analysis, which demonstrated that MSR1 overexpression in RAW264.7 cells promoted M2-like activation. There was no significant difference in OCR or polarization phenotype of MSR1-overexpressing RAW264.7 cells without co-culturing (Figures 5B, D, F, and H and Figures S5A-D).

These data suggested that MSR1 played a key role in macrophage mitochondrial OXPHOS and M2-like activation in the co-culture system.

### Potential regulatory role of macrophage MSR1 in PI3K/AKT/GSK3β/β-catenin signaling in the co-culture system

**Figure 6:**
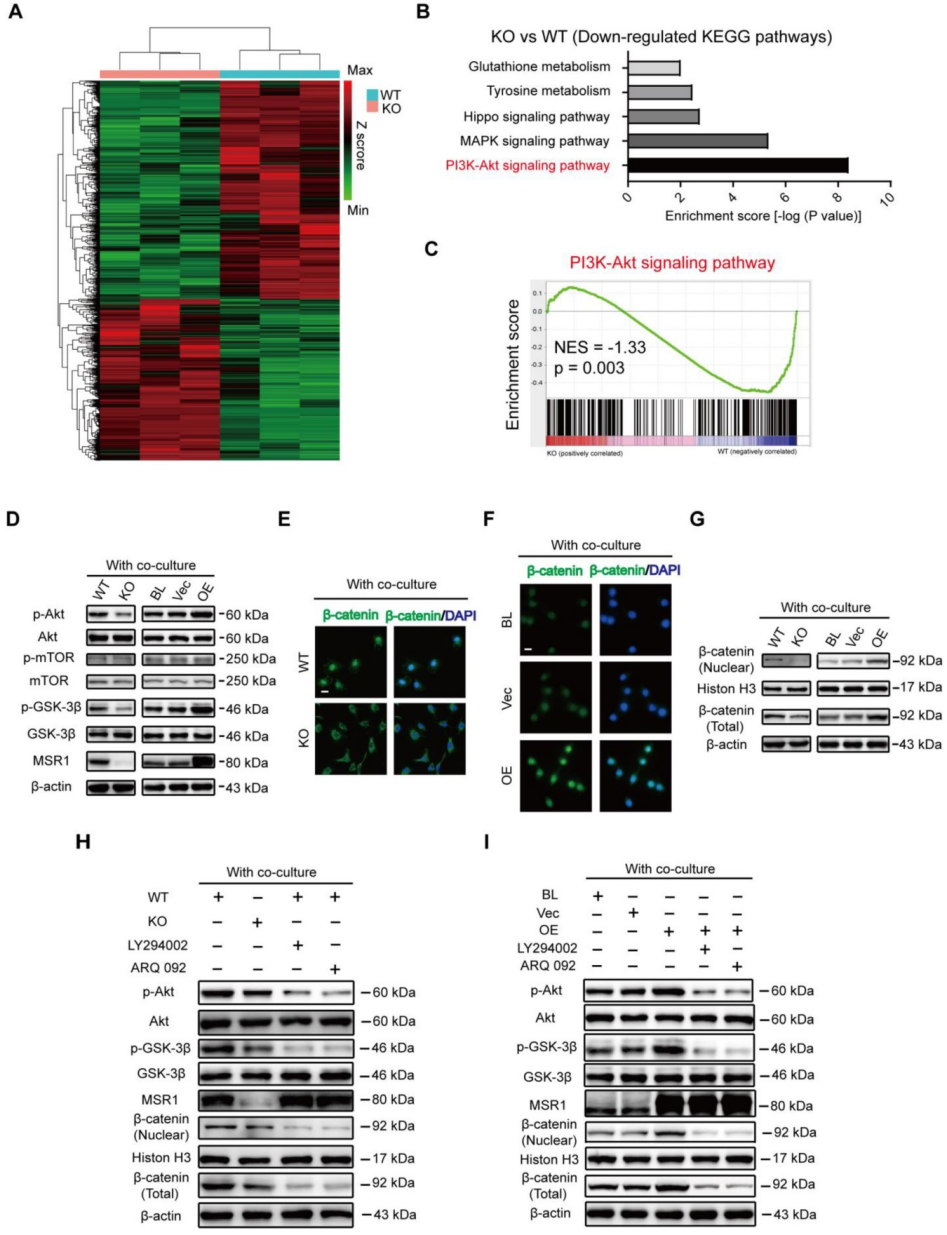
** MSR1 activates macrophage PI3K/AKT/GSK3β/β-catenin signaling in the co-culture system** (A) Heat map of DEGs of MSR1 KO or WT BMDMs after co-culturing with BMSCs. Green and red colors represent low and high expression values, respectively. (B) Representative down-regulated KEGG pathway categories affected by MSR1 depletion in macrophages. (C) Gene set enrichment analysis (GSEA) was used to identify the distribution of genes in the PI3K/AKT pathway gene set of MSR1 KO and WT groups. NES, normalized enrichment score. (D) Immunoblot images showing the effect of MSR1 KO or overexpression on the expression of p-AKT/AKT, p-mTOR/mTOR, and p-GSK3β/GSK3β after co-culturing with BMSCs. (E and F) Distribution of β-catenin (green) in MSR1 WT and MSR1 KO macrophages (E), and MSR1 BL, Vec, and overexpressing RAW264.7 cells (F) were analyzed by IF staining. The cell nuclei were stained with DAPI (blue fluorescence), Scale bars: 50 μm. (G) Immunoblot images showing the role of MSR1 KO or OE on the expression of β-catenin from nuclear and whole-cell lysates. (H) Altered protein expression levels of p-AKT/AKT, p-GSK3β/GSK3β, β-catenin (nuclear), and β-catenin (total) were detected using Western blotting in MSR1 KO and WT macrophages in the co-culture system, or in MSR1 WT macrophages treated with LY294002 (an inhibitor of PI3K), ARQ 092 (an inhibitor of AKT) before co-culture. (I) Protein expression levels of p-AKT/AKT, p-GSK3β/GSK3β, β-catenin (nuclear), and β-catenin (total) were detected using Western blotting in MSR1 BL, Vec and OE RAW264.7 cells in the co-culture system, or MSR1 OE RAW264.7 cells treated with LY294002 (an inhibitor of PI3K), ARQ 092 (an inhibitor of AKT) before co-culture.

To explore the mechanism related to the functions of pro-osteogenic differentiation and M2-like activation by MSR1, we performed transcriptome analysis by high-throughput RNA sequencing (RNA-Seq) using 3 biological replicates of MSR1 WT and KO macrophages after co-culturing with BMSCs. DEGs in this study were defined as genes with fold changes > 1.5 and p < 0.05. As illustrated in Figure 6A, 669 DEGs were upregulated while 902 DEGs were downregulated in MSR1-deficient BMDMs compared to control BMDMs. KEGG pathway analysis showed that the PI3K-AKT signaling pathway was markedly inhibited in MSR1 KO macrophages compared to the control group (Figure 6B). GSEA was also used to show the distribution of genes in different gene set pathways. The results revealed that genes related to the PI3K-AKT signaling pathway were significantly enriched in the MSR1 WT group, suggesting a potential regulatory role of MSR1 in the PI3K-AKT signaling pathway (Figure 6C). Activated PI3K-AKT signaling has been recognized as an essential step towards M2-like polarization and a negative regulator of TLR and NF-κB pathways in macrophages 18. Moreover, the PI3K/AKT/GSK3β pathway was shown to be activated during MSR1-mediated peritoneal macrophage spreading following adhesion to malondialdehyde-modified proteins 40. As indicated in Figure 6D, MSR1 KO decreased the level of phosphorylated AKT (Ser473) and GSK3β (Ser9), and overexpression of MSR1 positively influenced phosphorylated AKT and GSK3β in the co-culture system. However, neither phosphorylated AKT nor phosphorylated GSK3β was altered without co-culturing (Figure S6A). Importantly, the phosphorylated protein level of mTOR (Ser2448), another PI3K/AKT signaling substrate, was not regulated by MSR1 after co-culturing with BMSCs (Figure 6D).

PI3K/AKT pathway modulates many cellular functions through the inhibition of GSK3β (phosphorylated at position Ser9), which allows β-catenin to stabilize and translocate into the nucleus for gene transcription 18-20. After co-culturing with BMSCs, IF staining exhibited less nuclear localization of β-catenin (green) in MSR1 KO macrophages (Figure 6E). Additionally, the nuclear localization of β-catenin (green) was more pronounced in MSR1-overexpressing RAW264.7 cells (Figure 6F). Western blotting was performed to detect the expression and distribution of β-catenin. As depicted in Figure 6G, the total amount of β-catenin and nuclear β-catenin were both affected by MSR1. However, that total and nuclear localization of β-catenin was not regulated by MSR1 without co-culturing as indicated by IF and Western blotting (Figures S6B-D). To further confirm that the PI3K/AKT/GSK3β pathway is involved in MSR1-mediated stabilization and nuclear localization of β-catenin, small molecule inhibitors targeting PI3K (LY294002) or AKT (ARQ 092) were used. As shown in Figure 6H, AKT and GSK3β signaling was inhibited by LY294002 and ARQ 092 in MSR1 WT macrophages co-cultured with BMSCs. As expected, both total and nuclear-localized β-catenin decreased after treatment with LY294002 or ARQ 092, as indicated by the weaker band seen in Figure 6H. Similar results were observed in MSR1-overexpressing RAW264.7 cells treated with LY294002 or ARQ 092 before co-culturing (Figure 6I). These results were consistent with our RNA sequence data, which indicated that macrophage MSR1 could be a major regulator of PI3K/AKT/GSK3β/β-catenin signaling pathway in the co-culture system.

### MSR1-mediated PI3K/AKT/GSK3β/β-catenin signaling activation in macrophages is important for pro-osteogenic differentiation

**Figure 7:**
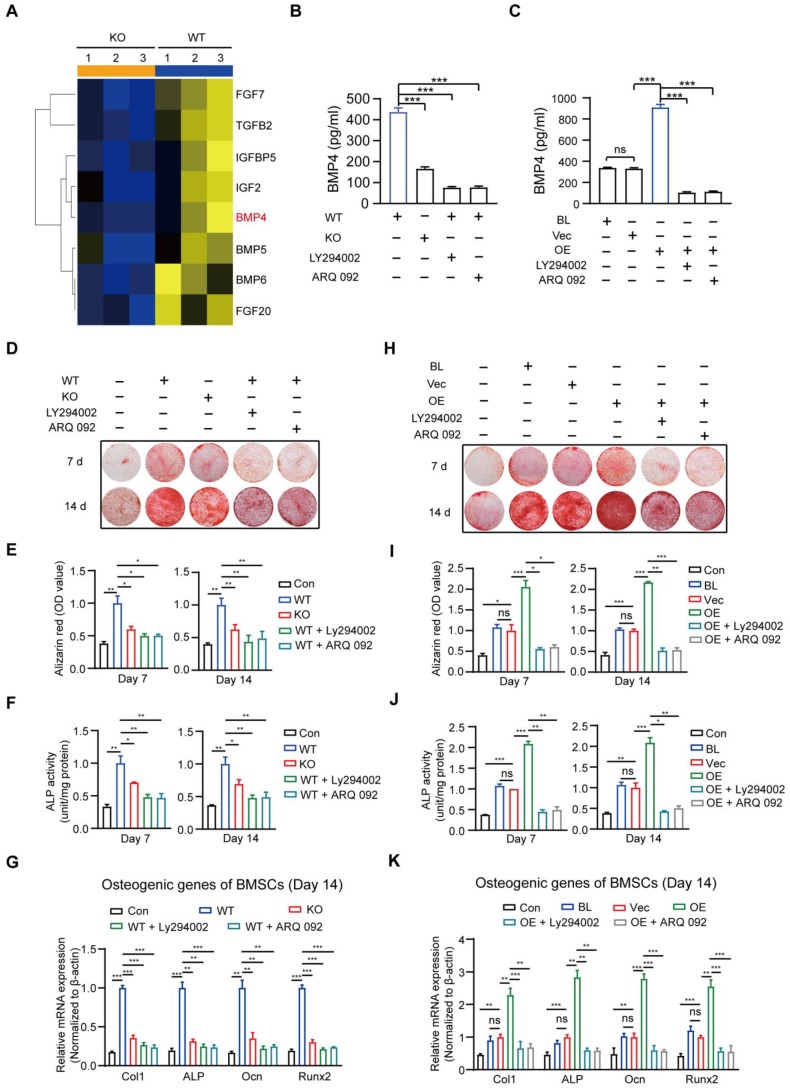
** MSR1-activated macrophage PI3K/AKT/GSK3β/β-catenin signaling promotes osteogenic differentiation of BMSCs.** (A) Heat map of several genes encoding molecules involved in BMSC osteogenic differentiation was performed based on the results of RNA sequencing (MSR1 KO vs. WT). Blue and yellow colors represent low and high expression values, respectively.(B) The amount of secreted BMP4 in 24-h in the serum-free medium by MSR1 WT and MSR1 KO macrophages after co-culture, or MSR1 WT macrophages treated with LY294002 or ARQ 092 before co-culture was assessed by ELISA. Values are expressed as mean ± SD, ***p < 0.001. (C) The amount of secreted BMP4 in 24-h serum-free MSR1 BL, Vec, and OE RAW264.7 cells after co-culture, or MSR1 OE RAW264.7 cells treated with LY294002, ARQ 092 before co-culture was determined by ELISA. Values are expressed as mean ± SD, ***p < 0.001. (D-F) In the co-culture system, knockout of MSR1 or inhibition of PI3K/AKT/GSK3β/β-catenin signaling in macrophages impaired pro-osteogenic differentiation of BMSCs as observed by AR staining (D). Quantitative evaluation of AR staining results (E) and ALP activities (F) on day 7 and 14 was performed. BMSC without co-culture was used as the Con group. Values are expressed as mean ± SD, *p < 0.05, **p < 0.01. (G) mRNA expression levels of osteogenic biomarkers (Col1, ALP, Ocn and Runx2) in osteogenic differentiated BMSCs on day 14 were detected by qPCR in different groups. β-actin was used as an internal control. Values are expressed as mean ± SD, **p < 0.01, ***p < 0.001. (H-J) Inhibition of PI3K/AKT/GSK3β/β-catenin signaling in MSR1 OE RAW264.7 cells in the co-culture system decreased osteogenic differentiation of BMSCs as observed by AR staining (H). Quantitative evaluation of AR staining results (I) and ALP activities (J) on day 7 and 14 was performed. Values are expressed as mean ± SD, *p < 0.05, **p < 0.01, ***p < 0.001, ns indicates no significance. (K) mRNA expression levels of Col1, ALP, Ocn and Runx2 in osteogenic differentiated BMSCs on day 14 detected by qPCR in the indicated groups. β-actin was used as an internal control. Values are expressed as mean ± SD, **p < 0. 01, ***p < 0.001, ns indicates no significance.

Cytokines are reported to promote osteogenic differentiation 41, 42; therefore, we attempted to identify the cytokines implicated in macrophage MSR1-mediated osteogenic differentiation of BMSCs. As indicated in Figure 7A and Figure S7A, based on the results of RNA-seq, eight significantly decreased pro-osteogenic differentiation cytokines (FGF7, TGFB2, IGFBP5, IGF2, BMP4, BMP5, BMP6, and FGF20) were identified in MSR1 KO macrophages after co-culturing with BMSCs 43-48. The mRNA expression patterns of the cytokines mentioned above in different groups were further tested using qPCR (Figure S7B). BMP4 was chosen (because of its highest value of fold-change) to explore the variable protein levels in different groups by ELISA. As shown in Figures 7B and C, secretion of BMP4 could be significantly blocked by MSR1 KO and small molecule inhibitors (LY294002 or ARQ 092). Besides, Alizarin Red staining and subsequent quantitative analysis confirmed that the pro-osteogenic differentiation effect of MSR1 in macrophages was modulated by PI3K/AKT/GSK3β/β-catenin signaling (Figures 7D-I). ALP activities and qPCR analysis of osteogenic marker genes (Col1, ALP, Ocn and Runx2) from BMSCs in different groups also supported this conclusion (Figures 7F-K, and Figures S7C and D).

Consistent with the previous results, macrophage MSR1-mediated PI3K/AKT/GSK3β pathway might contribute to the pro-osteogenic differentiation of BMSCs in the co-culture system.

### PGC1α is a target gene of MSR1-activated PI3K/AKT/GSK3β/β-catenin pathway to promote OXPHOS in macrophages

**Figure 8:**
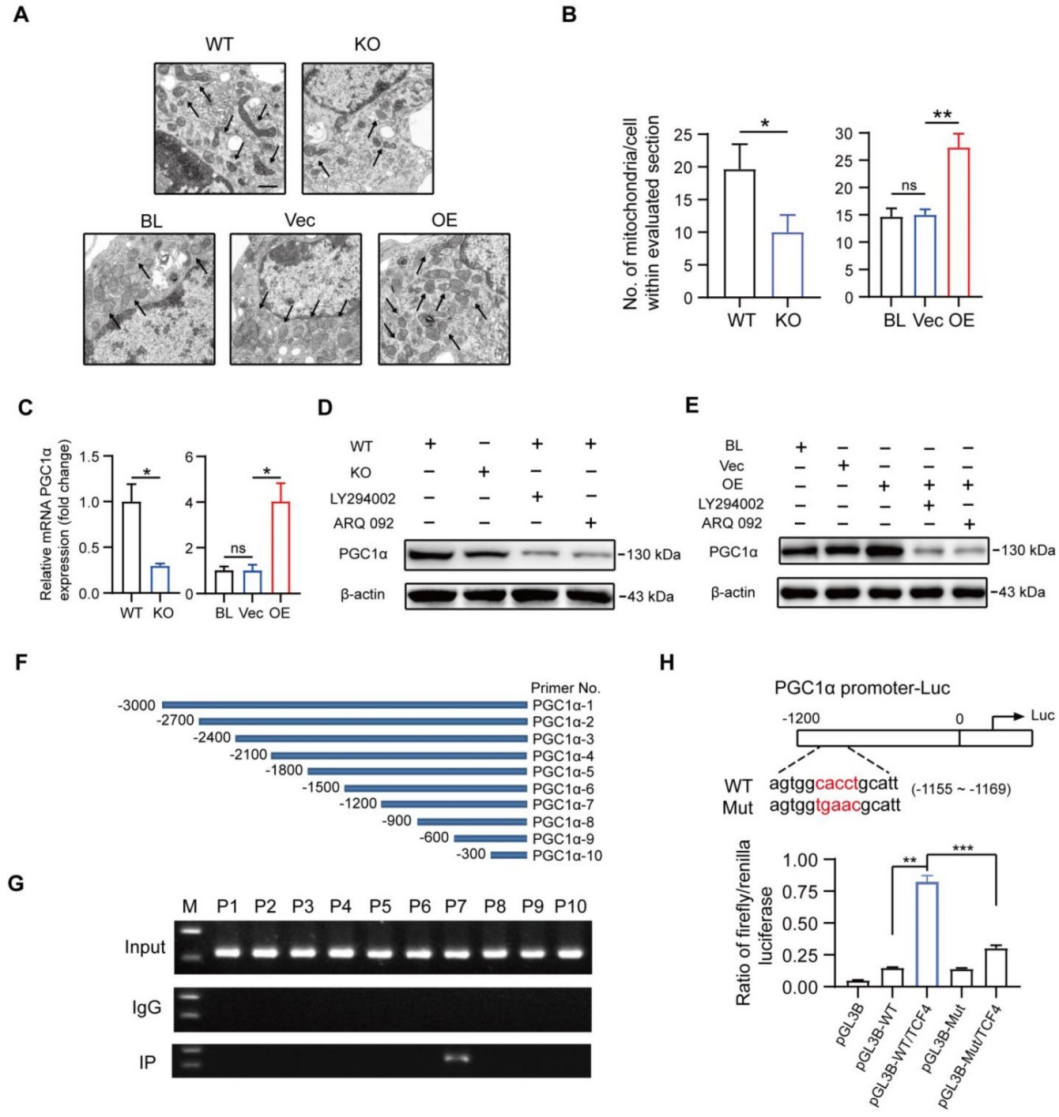
** Macrophage MSR1 regulates the expression of PGC1α in the co-culture system.** (A) The number of mitochondria per cell in different groups was evaluated by transmission electron microscopy. Black arrows indicate mitochondria. Scale bars: 500 nm. (B) Quantification of the number of mitochondria per cell in the imaged section. Values are mean ± SD, *p < 0.05, **p < 0.01, ns indicates no significance. (C) mRNA expression levels of PGC1α in MSR1 WT and MSR1 KO macrophages, and MSR1 BL, Vec, and MSR1 OE RAW264.7 cells were determined by qPCR. Values are mean ± SD, *p < 0.05, ns indicates no significance. (D) Altered protein expression level of PGC1α was detected by Western blotting in MSR1 KO macrophages in the co-culture system, or MSR1 WT macrophages treated with LY294002, ARQ 092 before co-culture. (E) Immunoblot images of the protein expression level of PGC1α in MSR1 BL and Vec RAW264.7 cells in the co-culture system, or MSR1 OE RAW264.7 cells treated with LY294002, ARQ 092 before co-culture. (F) Diagram depicting 10 pairs of primers in the promoter region of PGC1α. (G) ChIP assay was performed to confirm the potential TCF-binding site in the PGC1α promoter region in macrophages. Immunoprecipitated DNA was amplified with a series of primers covering the 3000 bp sequence upstream from PGC1α transcription start site. (H) Luciferase reporter assay was performed using macrophages after transfecting the wild-type and mutant PGC1α promoter (mutation site: red). Values are mean ± SD, **p < 0.01, ***p < 0.001.

It has previously been demonstrated that OXPHOS mediated the polarization and function of M2-like macrophages 15, 16. Additionally, mitochondrial biogenesis and functions were reported to be involved in activated PI3K/AKT pathway through GSK3β inhibition, but the underlying specific mechanisms in macrophages remained unclear 17, 18. As shown in Figures 8A and B, the number of mitochondria was significantly decreased in MSR1 KO macrophages after co-culturing with BMSCs, but an increased number of mitochondria could be observed in co-cultured MSR1-overexpressing RAW264.7 cells. Both RNA-Seq and qPCR data suggested a positive correlation between the mRNA expression of MSR1 and PGC1α, a key mediator of mitochondrial biogenesis (Figure 8C and Figure S7E) 49, 50. Thus, we next investigated whether MSR1-mediated PI3K/AKT/GSK3β/β-catenin pathway controlled the expression of PGC1α protein.

As indicated in Figure 8D, deletion of MSR1 and inhibition of PI3K/AKT/GSK3β/β-catenin signaling decreased the expression of PGC1α. Moreover, blocking the PI3K/AKT/GSK3β/β-catenin pathway in MSR1-overexpressing RAW264.7 cells reversed the up-regulated expression of PGC1α (Figure 8E). GSK3β inactivation is known for causing increased accumulation of β-catenin in the cytoplasm and its subsequent translocation into the nucleus for gene transcription. To demonstrate whether TCF4, a classic nuclear partner of β-catenin, directly regulated the expression of PGC1α, we first constructed 10 pairs of primers in the promoter region of PGC1α (Figure 8F). When the ChIP assay was performed, the results revealed that TCF4 had a binding site (-1200 to -900 bp upstream of the transcription start site) in BMDMs (Figure 8G). Furthermore, luciferase reporter plasmids containing about 1200 bp of the wild-type and mutant PGC1α promoters were constructed. The results of dual-luciferase reporter assay indicated that the transcriptional activity of the PGC1α promoter was significantly enhanced by TCF4, and decreased by a mutation in the PGC1α promoter (Figure 8H).

These results suggested that MSR1-mediated PI3K/AKT/GSK3β/β-catenin pathway enhanced OXPHOS by up-regulating PGC1α expression in macrophages after co-culturing with BMSCs. These findings explain our previous observations that MSR1 contributes to the M2-like polarization phenotype in a co-culture system.

### Transplantation with MSR1 from WT bone marrow improves the impaired intramembranous ossification in MSR1 KO mice

**Figure 9:**
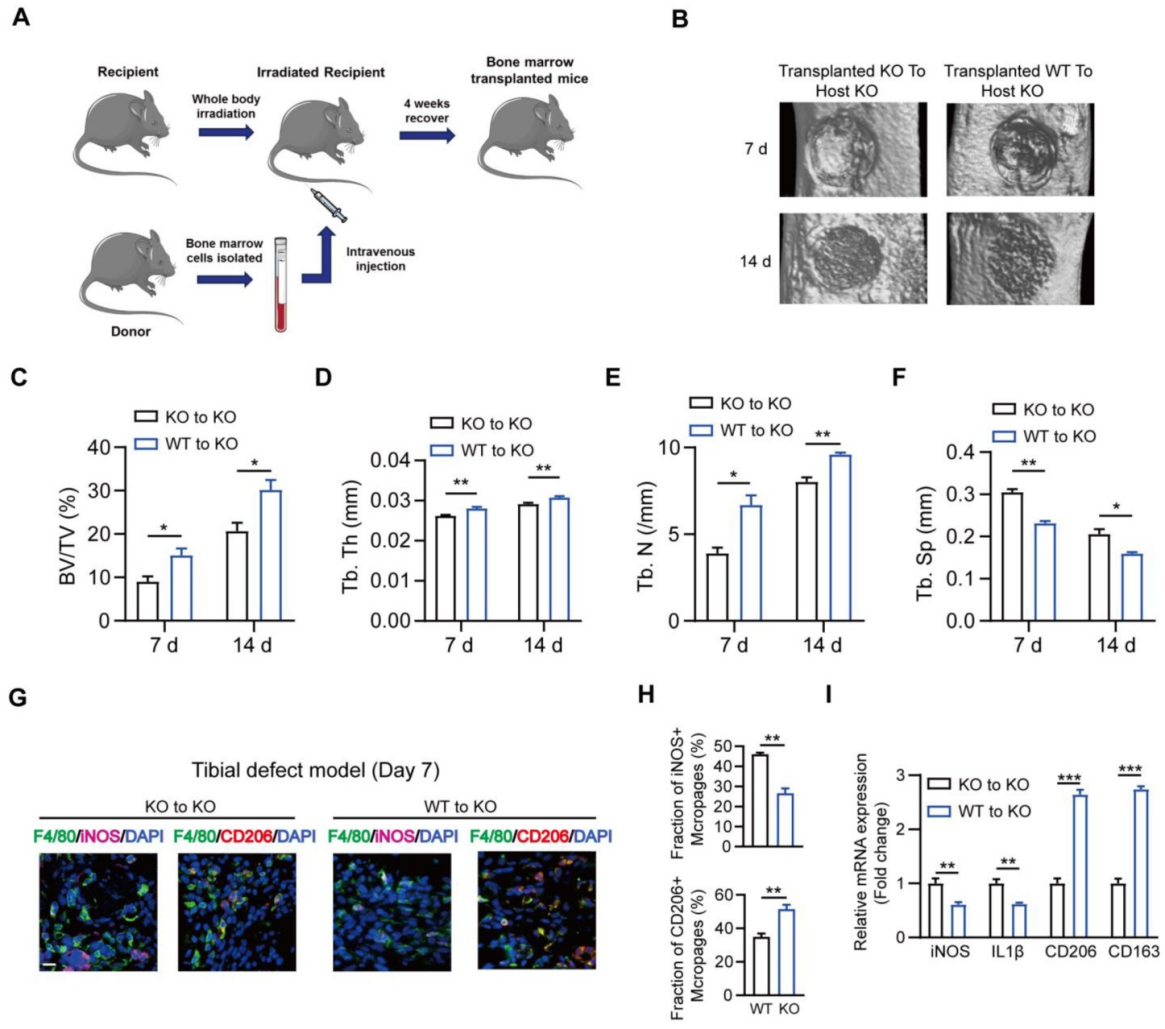
** Substitution of MSR1 KO bone marrow with MSR1 WT bone marrow promotes intramembranous bone healing.** (A) Schematic representation of the main steps of the bone marrow transplant. (B) Representation of 3D images of injured tibiae from different transplanted mice (KO to KO vs. WT to KO) by micro-CT on day 7 or 14 post-surgery. (C-F) Quantification of BV/TV (%) (C), Tb. Th (mm) (D), Tb. N (/mm) (E) and Tb. Sp (mm) (F) in the defect region on day 7 and 14 post-surgery for different transplanted mice (KO to KO vs. WT to KO) (Values are expressed as mean ± SD, *p < 0.05). (G) Representative IF images of total macrophages (F4/80+), M1-like macrophages (iNOS+ F4/80+) and M2-like macrophages (CD206+ F4/80+) in facture tissues from the bone marrow of transplanted mice (transplanted MSR1 KO bone marrow to host KO mice and transplanted MSR1 WT bone marrow to host KO mice) on day 7 post-surgery in the tibial monocortical defect model. Nuclei were counterstained with DAPI (blue). Bar = 100 μm. (H) The iNOS+ and CD206+ macrophage fractions were determined from different transplanted mice (KO to KO vs. WT to KO) on day 7 post-surgery of the tibial monocortical defect model (Values are expressed as mean ± SD, *p < 0.05, **p < 0.01). (I) mRNA expression levels of macrophage marker genes (M1-like: iNOS and IL-1b, M2-like: CD206 and CD163) in fracture tissues from different transplanted mice on day 7 in the tibial monocortical defect model (K) (*p < 0.05, **p < 0.01, ***p < 0.001).

To further verify the association between myeloid MSR1 and IO, bone marrow transplantation experiments were performed (Figure 9A). Four weeks after substituting MSR1 KO bone marrow with MSR1 KO or MSR1 WT bone marrow, a standardized tibial monocortical defect model was obtained. As shown in Figures 9B-F, and Figure S8A, enhanced IO was demonstrated in MSR1 KO mice transplanted with MSR1 WT bone marrow compared with those transplanted with MSR1 KO bone marrow. Also, irradiated MSR1 KO mice reconstituted with bone marrow from MSR1 WT mice up-regulated the fraction of CD206^+^ macrophages and down-regulated the fraction of iNOS^+^ macrophages (Figures 9G and H, and Figure S8B). A reversed callus mRNA expression level of M1-like markers (iNOS and IL1β) and M2-like markers (CD206 and CD163) were also observed in MSR1 KO mice transplanted with MSR1 WT bone marrow by qPCR (Figure 9I). These results supported the notion that MSR1 was not only responsible for the changes in the IO but also affected the infiltrated M2-like macrophages.

## Discussion

**Figure 10:**
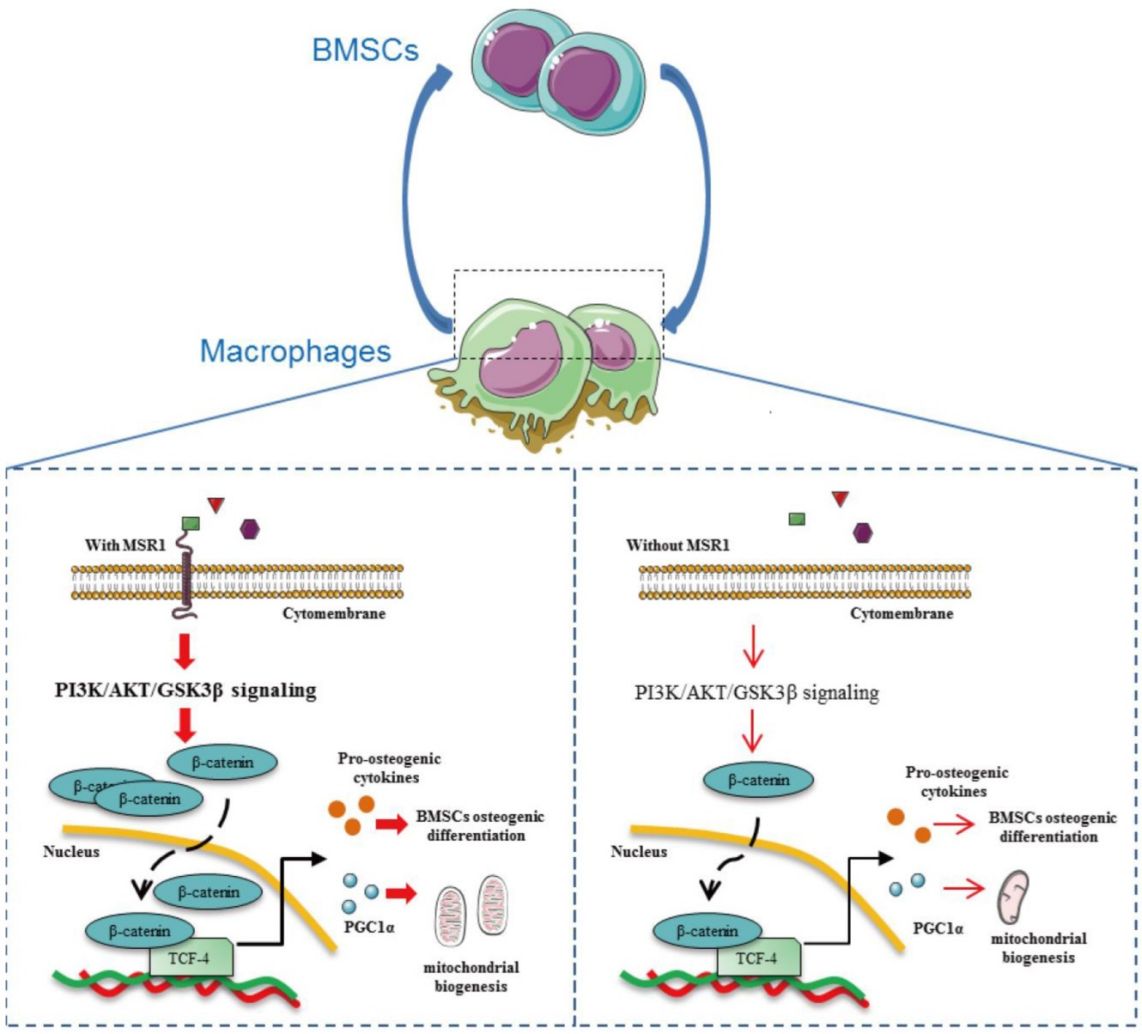
** Macrophage MSR1 is essential for maintaining M2-like activation and promoting BMSC osteogenic differentiation.** In the communication between macrophages and BMSCs, MSR1 plays a crucial role in maintaining M2-like polarization and facilitating BMSC osteogenic differentiation. MSR1-mediated activation of PI3K/AKT/GSK3β/β-catenin signaling promotes BMSC osteogenic differentiation. Notably, PGC1α is regulated by MSR1-mediated PI3K/AKT/GSK3β/β-catenin signaling in the macrophages. Increased PGC1α promotes oxidative phosphorylation by enhancing mitochondrial biogenesis to maintain M2-like activation in macrophages.

It has been shown that MSR1 acts as a protective receptor in macrophages and attenuates disease progression 21. Although published data also suggest that MSR1 contributes to the pathophysiology of certain diseases, little is known about its role in the process of IO 21. In the present study, we demonstrated that MSR1, a membrane receptor, contributes to the activation of the PI3K/AKT/GSK3β pathway by enhancing the nuclear translocation of β-catenin. MSR1-mediated PI3K/AKT/GSK3β/β-catenin signaling not only promotes BMSC osteogenic differentiation but also facilitates mitochondrial biogenesis and OXPHOS by up-regulating the expression of PGC1α, which participates in the M2-like activation (Figure 10).

Recent studies conducted in the co-culture system and animal models have pointed out that macrophage-BMSC cross-talk had a great impact on bone regeneration during fracture repair 10, 51. However, the underlying mechanisms of this communication between BMSCs and macrophages were not characterized. Macrophage-derived chemokines (such as CCL2, SDF-1, and CXCL8) and osteoinductive factors (such as BMP2, OSM, and PGE2) could regulate the recruitment and osteogenic differentiation of BMSCs 10. Depletion of macrophages led to an impaired IO as indicated by the reduced osteogenic differentiation ability of BMSCs and decreased deposition of woven bone 8-10. Our study shows that macrophage MSR1 contributes to IO *in vivo* and pro-osteogenic differentiation of BMSCs in a co-culture system. Mechanistically, in this co-culture system, macrophage MSR1-mediated PI3K/AKT/GSK3β/β-catenin signaling could be involved in producing BMP4.

BMSCs have been shown to reciprocally modulate macrophage polarization phenotype 52-54. Several studies have revealed that BMSCs were able to suppress M1-like polarization and promote M2-like polarization 52, 53. However, the potential mechanisms by which BMSCs influence polarization of macrophages remained unclear. In the present study, we found that lack of MSR1 led to a reduced OXPHOS and M2-like polarization phenotype. Mechanistically, when co-cultured with BMSCs, macrophage MSR1 regulated the expression of PGC1α via PI3K/AKT/GSK3β/β-catenin pathway to maintain M2-like polarization. PGC1α is a central regulator of mitochondrial biogenesis and OXPHOS, and current findings highlight the key role of metabolic cascades in macrophage activation and function 37, 38. M1-like macrophages take advantage of glycolysis for phagocytosis and killing, whereas M2-like macrophages rely on OXPHOS for sustained energy production for tissue repair 37, 38. We detected a novel role of MSR1 in mediating the OXPHOS of macrophages in the co-culture system. Thus, manipulating M2-like macrophages through MSR1 may represent a new therapeutic approach for bone regeneration.

In this study, we obtained BMDMs from MSR1 KO and WT mice to investigate the role of MSR1. We performed gain of function experiments by overexpressing MSR1 in RAW264.7 cells and demonstrated novel functions of MSR1, such as enhanced BMSC osteogenic differentiation and facilitation of macrophage OXPHOS. We also confirmed that macrophage MSR1 activated PI3K/AKT/GSK3β/β-catenin signaling in a co-culture system.

Although we evaluated IO using MSR1 KO mice, it may still be of somewhat limited value. Accurate determination of the role of MSR1 *in vivo* requires further investigations using conditional KO in mice, specifically targeting macrophages. Other directions that need to be pursued in the future include identification of the specific ligands associated with the paracrine effects of BMSCs or with the autocrine effects of macrophages. Given the high plasticity of macrophages, it is plausible that their polarization status could also be controlled by other metabolic pathways involving amino acids, lipids, and iron 55. The cross-talk between the macrophages and BMSCs is complex, and other molecular components/pathways affected by MSR1 need further investigation. In this study, non-activated macrophages were used; whether MSR1 contributes to the immune-modulatory function of BMSCs in regulating M1-like and/or M2-like activated macrophages and the specific mechanisms involved in M1/M2 polarized macrophages requires further analysis. Moreover, whether MSR1 contributes to the endochondral bone formation and/or chondrogenesis is worthy to investigate in further study.

In conclusion, our study elucidated a previously unrecognized function of MSR1 that underlies promotion of IO during fracture repair and enhanced osteogenic differentiation of BMSCs in a co-culture system. This effect correlated with the activation of PI3K/AKT/GSK3β/β-catenin signaling to induce production of several osteoinductive factors. MSR1-activated PI3K/AKT/GSK3β/β-catenin pathway contributed to OXPHOS for M2-like polarization by increasing the expression of PGC1α.

## Supplementary Material

Supplementary figures and table.
