# Supplementary material for: Macrophage MSR1 promotes BMSC osteogenic differentiation and M2-like polarization by activating PI3K/AKT/GSK3β/β-catenin pathway
Source: Theranostics. 2020 Jan 1;10(1):17–35. doi: 10.7150/thno.36930 (PMC6929615; doi:10.7150/thno.36930)
Supplement: Supplementary file 1 — Supplementary figures and table. [file thnov10p0017s1.pdf]

A

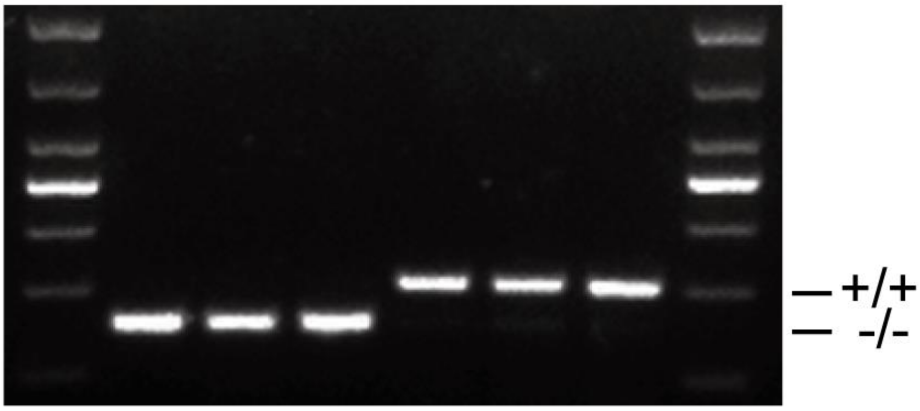

Figure S1

(A) Genotyping of MSR1 WT or MSR1 KO mice was confirmed by PCR of DNA samples from tail chips.

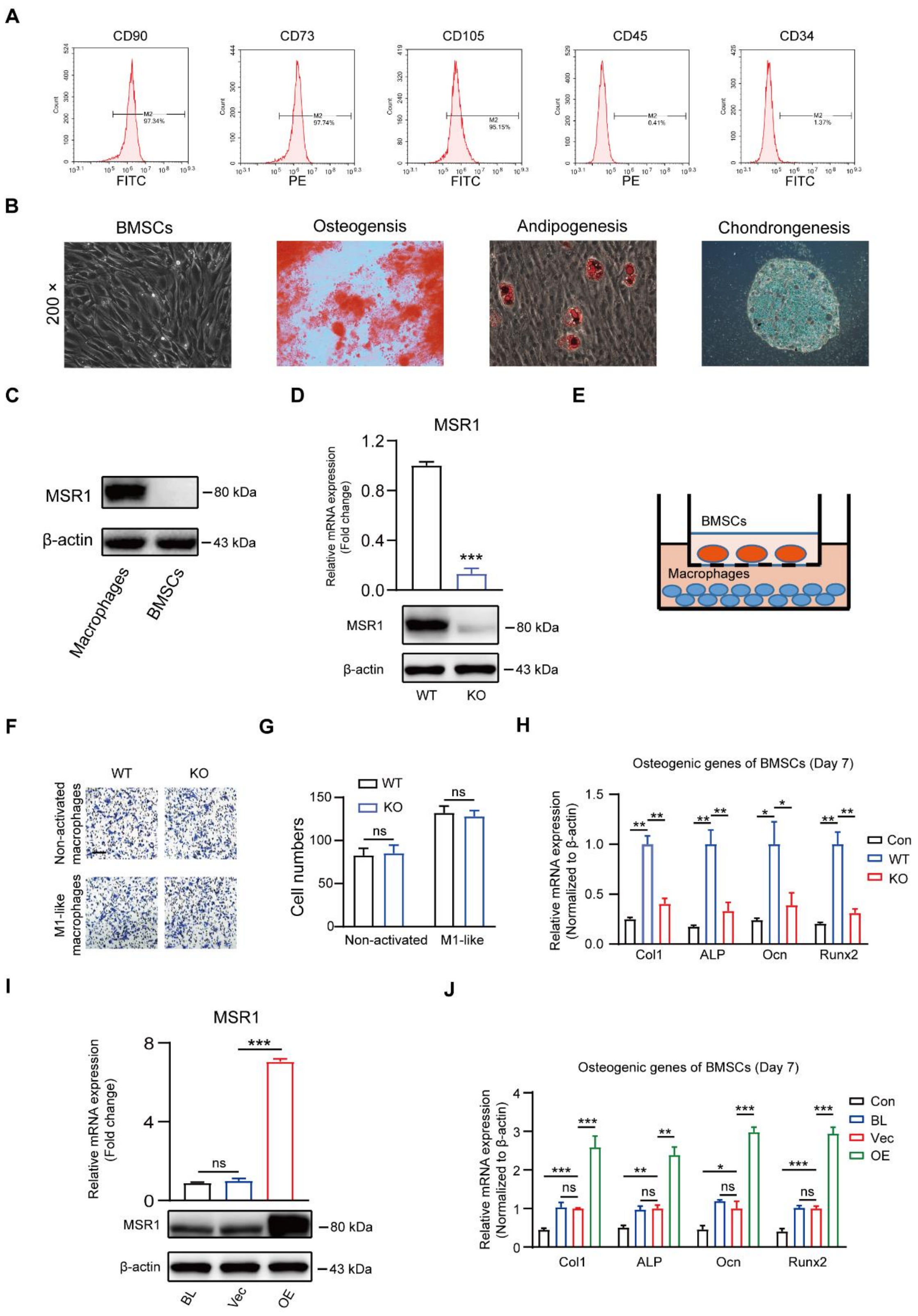

Figure S2

(A) Flow cytometry was used to identify the phenotype profile of BMSCs (positive makers: CD90, CD73 and CD105; negative makers: CD45, and CD34), and the

representative results are shown.

(B) Staining with alizarin red, oil red O, alcian blue was performed to test the osteogenic, adipogenic, and chondrogenic differentiation of BMSCs, respectively.

(C) Immunoblot images showing the expression patterns of MSR1 in BMDMs and BMSCs.

(D) The knockout efficiency of MSR1 in macrophages was confirmed by qPCR and Western blotting. Values are mean ± SD, ***$p < 0.001$.

(E) As shown in the schematic of co-culture cell migration assay, BMSCs were seeded in the upper chamber, and macrophages were cultured in the lower chamber.

(F and G) MSR1 did not affect the migration of BMSCs in vitro. Migration ability of BMSCs co-cultured with non-activated or LPS-activated BMDMs from MSR1 WT or KO mice was detected (F). Quantification of migrated cells (G). Values are mean ± SD, ns indicates no significance.

(H) mRNA expression levels of osteogenic marker genes (Col1, ALP, Ocn and Runx2) in osteogenic differentiation of BMSCs on day 7 using qPCR in different groups. β-actin was used as an internal control in this study (Values are mean ± SD, *$p < 0.05$, **$p < 0.01$).

(I) Overexpression efficiency of MSR1 in RAW264.7 cells was confirmed by qPCR and Western blotting. Values are mean ± SD, ***$p < 0.001$, ns indicates no significance.

(J) mRNA expression levels of Col1, ALP, Ocn and Runx2 in osteogenic differentiation of BMSCs on day 7 were detected by qPCR in different groups. β-actin

was used as an internal control (Values are mean ± SD, *p < 0.05, **p < 0.01, ***p < 0.001, ns indicates no significance).

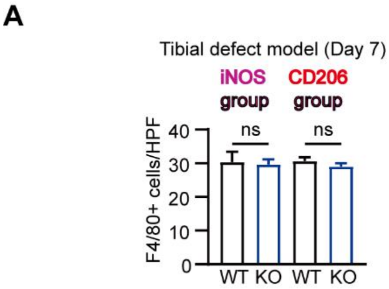

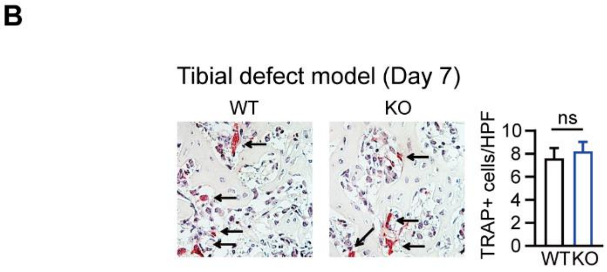

Figure S3

(A) Infiltration of F4/80+ macrophages was detected on day 7 post-surgery in the tibial monocortical defect model from MSR1 WT and KO mice, (Values are mean ± SD, ns indicates no significance). iNOS group indicates the samples stained with anti-F4/80 and anti-iNOS; the slides stained with anti-F4/80, and anti-CD206 denote the CD206 group.

(B) Tartrate-resistant acid phosphatase staining (TRAP) staining was used to detect the number of osteoclasts in fracture tissues of the tibial monocortical defect model on day 7 post-surgery (Values are mean ± SD, ns indicates no significance).

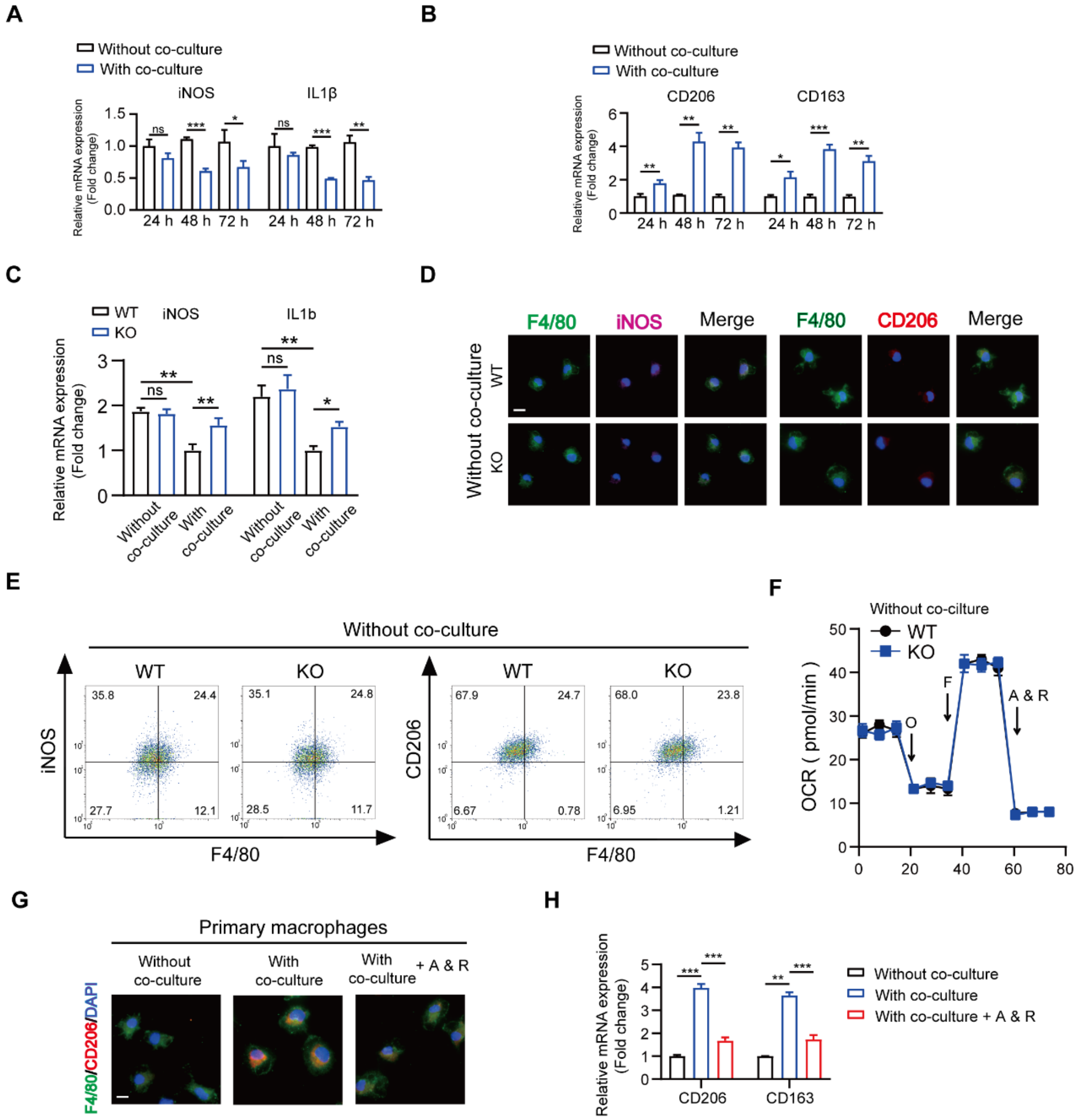

Figure S4

(A and B) mRNA expression levels of M1-like marker genes (iNOS and IL-1b) (A) and M2-like marker genes (CD206 and CD163) (B) in MSR1 WT BMDMs with or without co-culture at indicated time-points (24, 48, and 72 h) were detected by qPCR. Values are mean ± SD, *p < 0.05, **p < 0.01, ***p < 0.001, ns indicates no significance.

(C) mRNA expression levels of M1-like marker genes (iNOS and IL-1b) in MSR1 WT and KO macrophages with or without co-culture were detected by qPCR. Values

are mean ± SD, *p < 0.05, **p < 0.01, ns indicates no significance.

(D) IF staining results of MSR1 WT and KO macrophages without co-culture for M1 marker (iNOS) and M2 marker (CD206). Bar = 50 μm.

(E) Flow cytometry analysis of MSR1 WT or KO macrophages cultured alone. Dot plots represent F4/80 and iNOS staining of macrophages (left panel), and F4/80 and CD206 staining of macrophages (right panel).

(F) OCR of BMDMs in MSR1 WT or KO group without co-culture was detected using a Seahorse Bioscience XFp analyzer. O: Oligomycin, F: Carbonyl cyanide 4-(trifluoromethoxy)phenylhydrazone (FCCP), A&R: antimycin A/rotenone.

(G) IF staining results of MSR1 WT BMDMs in indicated groups for the M2-like marker (CD206). Bar = 50 μm.

(H) mRNA expression levels of M2 marker genes (CD206 and CD163) in MSR1 WT BMDMs in different groups were detected by qPCR. Values are mean ± SD, **p < 0.01, ***p < 0.001.

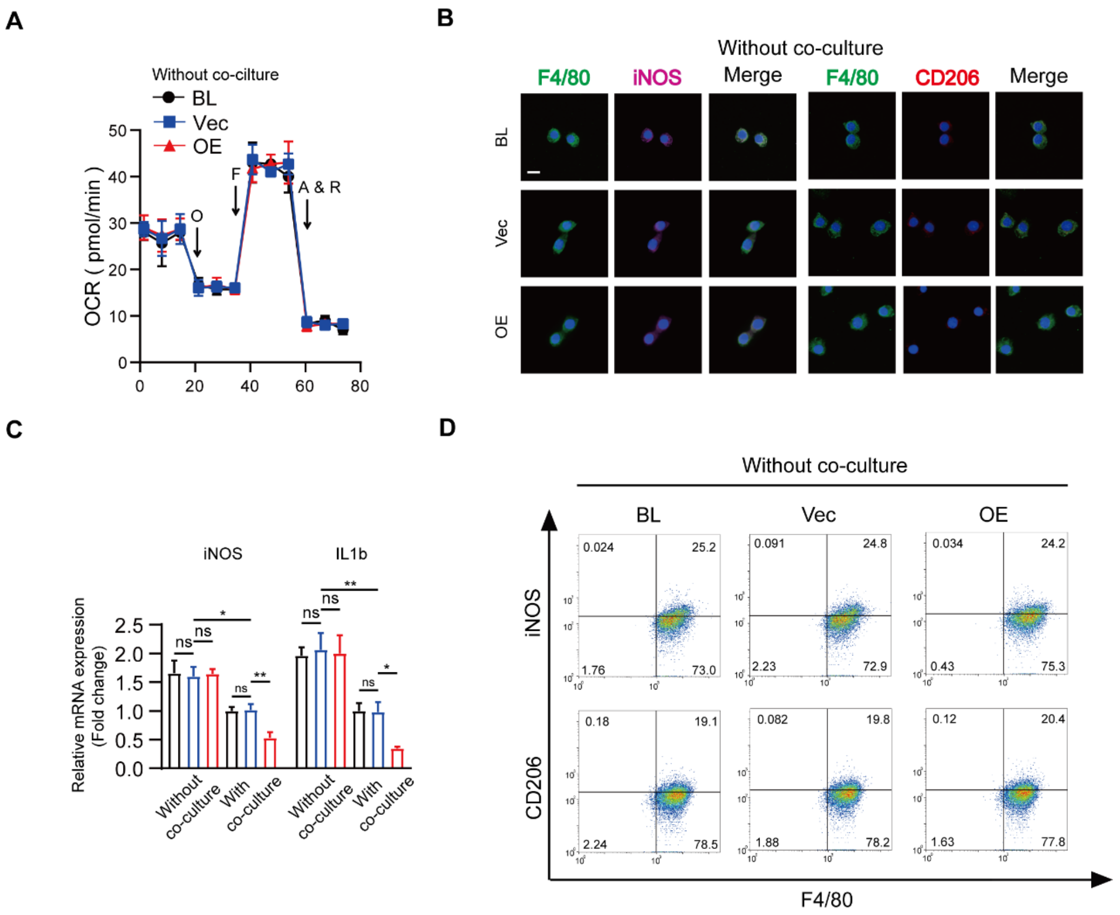

Figure S5

(A) OCR of blank (BL), vector (Vec) and MSR1-overexpression (OE) RAW264.7 cells without co-culture was evaluated using a Seahorse Bioscience XFp analyzer.

(B) IF staining results of BL, Vec, and MSR1-OE RAW264.7 cells without co-culture for the M1-like marker (iNOS) and M2-like marker (CD206). Bar = 50 μm.

(C) mRNA expression levels of M1-like macrophage marker genes (iNOS and IL-1b) in indicated groups were tested by qPCR. Values are mean ± SD, *p < 0.05, **p < 0.01, ns indicates no significance.

(D) Flow cytometry analysis of RAW264.7 cells from different groups without co-culture. Dot plots represent F4/80 and iNOS staining of RAW264.7 cells, and F4/80 and CD206 staining of RAW264.7 cells.

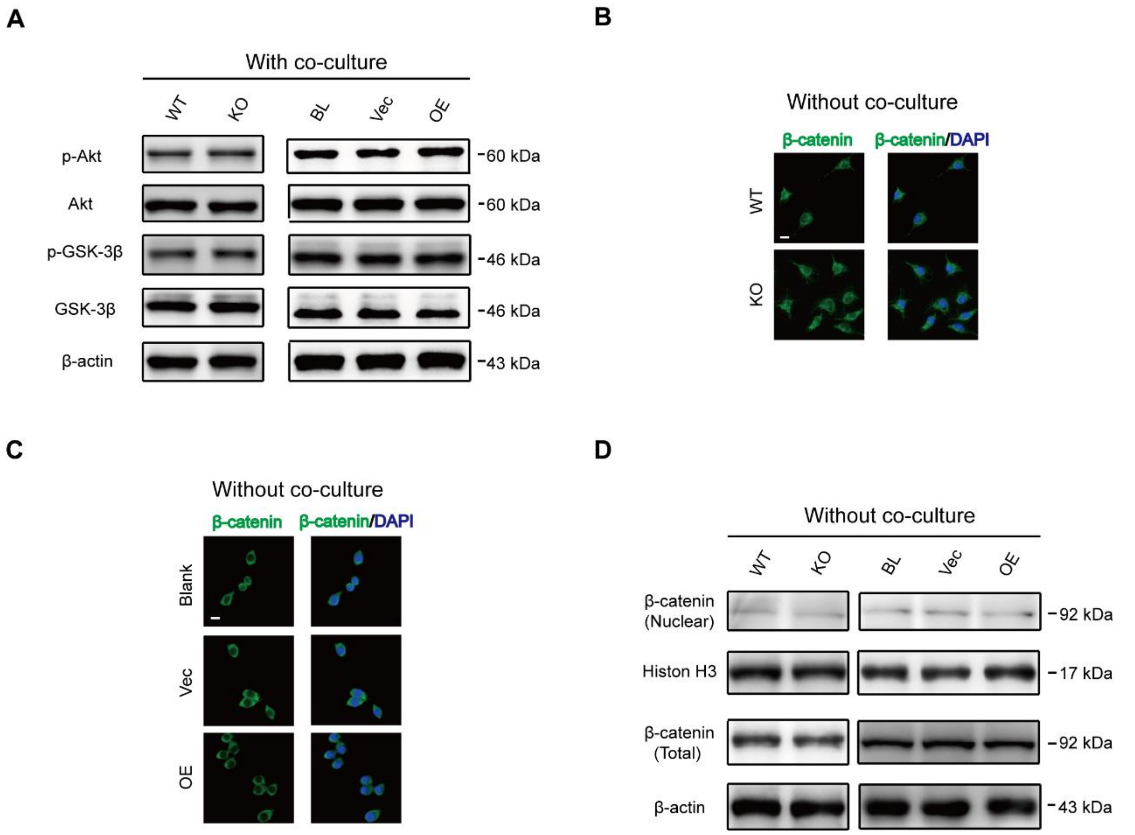

Figure S6

(A) Immunoblot images showing the effect of macrophage MSR1 KO or OE on the expression of p-AKT/AKT and p-GSK3β/GSK3β without co-culture.

(B and C) Distribution of β-catenin (green) in MSR1 WT and MSR1 KO BMDMs (B), and MSR1 BL, Vec, and OE RAW264.7 cells (C) without co-culture were analyzed by cell IF staining. The cell nuclei were stained with DAPI (blue fluorescence), Scale bars: 50 μm.

(D) Immunoblot images showing the role of MSR1 KO or OE cultured alone on the expression of β-catenin from nuclear and whole-cell lysates.

A

Gene	Log2 (Fold change)
igf2	-1.326
igfbp5	-0.997
bmp4	**-1.703**
bmp5	-0.963
bmp6	-1.653
fgf7	-0.746
fgf20	-1.582
tgfb2	-0.760

B

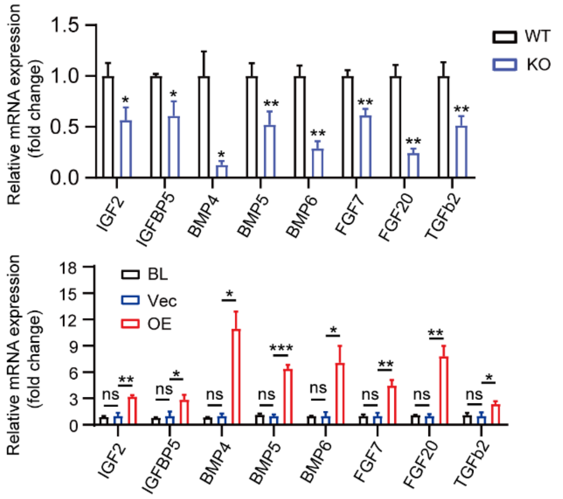

C

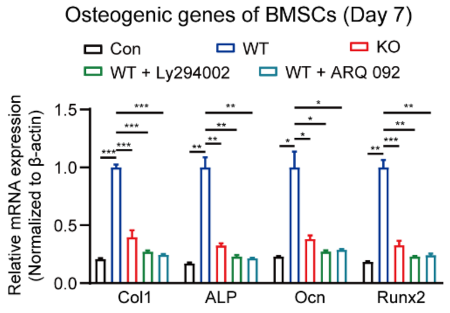

D

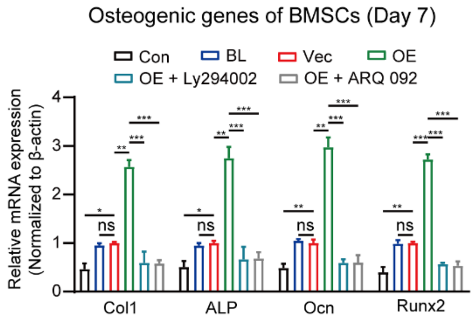

E

Mitochondrial biogenesis related genes

Gene	Log2 (Fold change)
ppargc1a (PGC1α)	-4.05
ppargc1b (PGC1β)	0.23
nrf1	-0.06
esrra	-0.06

Figure S7

(A) Log2 (fold change) value of several pro-osteogenic differentiation cytokines (FGF7, TGFB2, IGFBP5, IGF2, BMP4, BMP5, BMP6, and FGF20) based on RNA-sequencing.

(B) mRNA expression levels of osteoinductive factors (FGF7, TGFB2, IGFBP5, IGF2, BMP4, BMP5, BMP6, and FGF20) in MSR1 WT and MSR1 KO BMDMs (upper panel), and MSR1 BL, Vec, and MSR1 OE RAW264.7 cells (lower panel) were determined by qPCR. Values are mean ± SD, *p < 0.05, **p < 0.01, ***p < 0.001, ns

indicates no significance.

(C) mRNA expression levels of osteogenic markers (Col1, ALP, Ocn and Runx2) in osteogenic differentiated BMSCs at day 7 were detected by qPCR in different groups. β-actin was used as an internal control. Values are mean ± SD, *$p < 0.05$, **$p < 0.01$, ***$p < 0.001$.

(D) mRNA expression levels of Col1, ALP, Ocn and Runx2 in osteogenic differentiated BMSCs on day 7 were detected by qPCR in the indicated groups. β-actin was used as an internal control. Values are mean ± SD, *$p < 0.05$, **$p < 0.01$, ***$p < 0.001$, ns indicates no significance.

(E) Log2 (fold-change) values of mitochondrial biogenesis related genes (PGC1α, PGC1β, Nrf1 and Esrra) based on RNA-sequence.

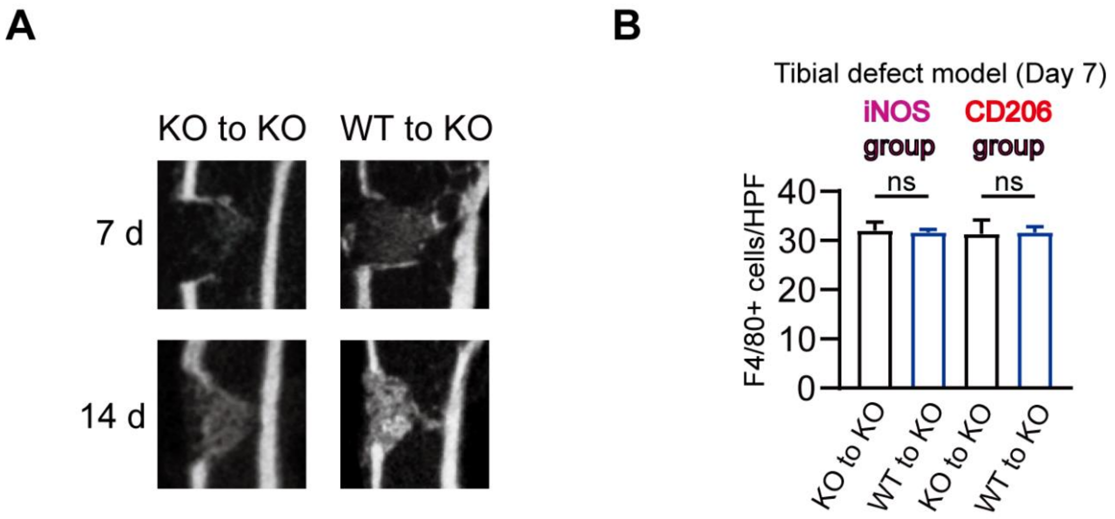

Figure S8

(A) Representative 2D coronal images of injured tiblae from different transplanted mice (KO to KO vs. WT to KO) on 7 or 14 days post-injury.

(B) The infiltration of F4/80+ macrophages in different groups were determined on 7 days post-surgery in tibial monocortical defect model from different transplanted mice (KO to KO vs. WT to KO) (Values are mean ± SD, ns indicates no significance). iNOS group indicates the samples stained with anti-F4/80 and anti-iNOS; the slides stained with anti-F4/80, and anti-CD206 denote the CD206 group.

Supplementary Table 1

The sequences of primers, wild-type and mutant PGC1α promoter region		
The primer sequences for qPCR		
Mouse	sense 5'-3'	antisense 5'-3'
MSR1	TGGAGGAGAGAATCGAAAGCA	CTGGACTGACGAAATCAAGGAA
RUNX2	AGAGTCAGATTACAGATCCCAGG	TGGCTCTTCTTACTGAGAGAGG
OCN	ATGAGCCCTCAGACTCCTC	CGGCCGTAGAGCGCCGATA
ALP	CCAACTCTTTTGTGCCAGAGA	GGCTACATTGGTGTTGAGCTTTT
Col1	CTGGCGGTTCAGGTCCAAT	TTCCAGGCAATCCACGAGC
iNOS	CAGGAGGAGAGAGATCCGATTTA	GCATTAGCATGGAAGCAAAGA
IL-1β	TGGAAAAGCGGTTTGTCTTC	TACCAGTTGGGGAACTCTGC
CD206	CTTCGGGCCTTTGGAATAAT	TAGAAGAGCCCTTGGGTTGA
CD163	GGTGGACACAGAATGGTTCTTC	CCAGGAGCGTTAGTGACAGC
PGC1α	TATGGAGTGACATAGAGTGTGCT	GTCGCTACACCACTTCAATCC
IGF2	GTGCTGCATCGCTGCTTAC	CGGTCCGAACAGACAAACTG

IGFBP5	CCCTGCGACGAGAAAGCTC	GCTCTTTTCGTTGAGGCAAAC C
Bmp4	TTGATACCTGAGACCGGGAA G	ACATCTGTAGAAGTGTCGCCT C
Bmp5	TTACTTAGGGGTATTGTGGGC T	TGAACGTGATTGTCTCCCAAG
Bmp6	GCGGGAGATGCAAAAGGAGA T	ATTGGACAGGGCGTTGTAGA G
FGF7	TGGGCACTATATCTCTAGCTT GC	GGGTGCGACAGAACAGTCT
FGF20	AGGATCACAGTCTCTTCGGTA TC	GTCATTCATCCCAAGGTACAG G
TGFB2	CTTCGACGTGACAGACGCT	GCAGGGGCAGTGTAAACTTAT T
β-actin	GGCTGTATTCCCCTCCATCG	CCAGTTGGTAACAATGCCATG T

The primer sequences for the ChIP assay		
	sense 5'-3'	antisense 5'-3'
P1	AATGTGTGGCCGAACACACTG TAGA	TTGTTAGTTGCTCACCAACCT TGGA
P2	TTTAAATTCTCTTGAGAAGAG	TGCTTAAAACAGCCACTCTGT

	CAAA	CCTC
P3	GATCATTAGCTTCATGGATGTGCTG	TAACTGATACTTTGGTTTCTCTTTG
P4	CCATCAGGATGCCAGGATTGCTTGA	TCCAAACATCAGAAAGGTATTATTC
P5	AGAGGACAGTTGTAGCAGTGAAGTA	TCTGACTTTATATAGTCAGTTACTA
P6	CTGAGAGAAGTCACCAATGTTTTCC	TAACCAACTCTCAGCACTTTCCATA
P7	TGTCCTCTGTCTGTAATGTCACAGG	CAGCTTAGCTACTCACCTGCCCCCA
P8	TTTCAGGGATGGCAGCAGCAATTGT	CTTTCTTTCTTTCTTTCTTTCTTTC
P9	AAAGAAACAAAGAAAGAAAGAAAGA	AAAACAGGCAAATAGCAAAGATCCC
P10	GGATGGAAAATAAATTTAAAAAAAA	AAAGCTATTAAAAAGTAGGCTGGGC

The sequence of wild-type and mutanted promoter region of PGC1α		
	wild-type	mutation
-1250 — -1201	gggttaagtctgagcacccaagtgttatggaaagtgctgagagttggtta	gggttaagtctgagcacccaagtgttatggaaagtgctgagagttggtta

-1200 —<br>-1151	tgtcctctgtctgtaatgtcacaggaaaaacagtg<br>g**cacct**gcattaccc	tgtcctctgtctgtaatgtcacaggaaaaacagtg<br>g**tgaac**gcattaccc
-1150 —<br>-1101	ctcattgactcaggaacgacaaaaaagtattagt<br>aagcaaagctcaagaa	ctcattgactcaggaacgacaaaaaagtattagt<br>aagcaaagctcaagaa
-1100 —<br>-1051	atgagtatctctgctgataccatttcagtgtttttcct<br>tcattccctgga	atgagtatctctgctgataccatttcagtgtttttcct<br>tcattccctgga
-1050 —<br>-1001	cattcttgatttcaaaaacaaactgtacagcccaa<br>ggcactagggttgga	cattcttgatttcaaaaacaaactgtacagcccaa<br>ggcactagggttgga
-1000 —<br>-951	gtccaatgtttattcaaaaaggcaccctgaagcc<br>atgaggaagactgtgc	gtccaatgtttattcaaaaaggcaccctgaagcc<br>atgaggaagactgtgc
-950 —<br>-901	tacatatgagaaaagaaataaggggtgggggc<br>aggtgagtagctaagctg	tacatatgagaaaagaaataaggggtgggggc<br>aggtgagtagctaagctg
-900 —<br>-851	tttcagggatggcagcagcaattgtattttctagc<br>atttgttttctggga	tttcagggatggcagcagcaattgtattttctagc<br>atttgttttctggga
-850 —<br>-801	gcctatgagatccacggaaagaatcatgagggg<br>gaacccaagagtctagg	gcctatgagatccacggaaagaatcatgagggg<br>gaacccaagagtctagg
-800 —<br>-751	gtgttgtggcttgcttgctttacaaggagcaaggc<br>aaactgcagtaacag	gtgttgtggcttgcttgctttacaaggagcaaggc<br>aaactgcagtaacag
-750 —<br>-701	tttaggagactgcattctctactgccaaggagac<br>agctgatttggggtag	tttaggagactgcattctctactgccaaggagac<br>agctgatttggggtag
-700 —<br>-651	agaaatttgtttagacctaaacaaatgtggcggttt<br>tgttgactaaacat	agaaatttgtttagacctaaacaaatgtggcggttt<br>tgttgactaaacat

-650 —<br>-601	ggaaagaaagaaagaaagaaagaaagaaaga<br>aagaaagaaagaaagaaag	ggaaagaaagaaagaaagaaagaaagaaaga<br>aagaaagaaagaaagaaag
-600 —<br>-551	aaagaaacaaagaaagaaagaaagaaaggaa<br>ggaaggaaggaagaaagga	aaagaaacaaagaaagaaagaaagaaaggaa<br>ggaaggaaggaagaaagga
-550 —<br>-501	agaaaggaaggaaggaaggaaggaaagaaa<br>ggagagagagaaagaaaatc	agaaaggaaggaaggaaggaaggaaagaaa<br>ggagagagagaaagaaaatc
-500 —<br>-451	gggggtgttgccttcaaacactcctctaataggg<br>agggaaaaaaaagaat	gggggtgttgccttcaaacactcctctaataggg<br>agggaaaaaaaagaat
-450 —<br>-401	ctcatgaaaatgtatcacatgaggagcgcttgctt<br>cagttccaagctgag	ctcatgaaaatgtatcacatgaggagcgcttgctt<br>cagttccaagctgag
-400 —<br>-351	tctggggctacttggaaaccatttcttaaagcaca<br>cacattttaggcaag	tctggggctacttggaaaccatttcttaaagcaca<br>cacattttaggcaag
-350 —<br>-301	ggtgtagttactgtgtcagtaacaggggatctttg<br>ctatttgcctgtttt	ggtgtagttactgtgtcagtaacaggggatctttg<br>ctatttgcctgtttt
-300 —<br>-251	ggatggaaaataaatttaaaaaaaaaagattgca<br>ggagatttgagttatt	ggatggaaaataaatttaaaaaaaaaagattgca<br>ggagatttgagttatt
-250 —<br>-201	atgtgagcagggctccggtttagagttggtggca<br>ttcaaagctggcttca	atgtgagcagggctccggtttagagttggtggca<br>ttcaaagctggcttca
-200 —<br>-151	gtcacagtgtgatgcttgaagcctcccaaaggcc<br>aagtgtttccttttct	gtcacagtgtgatgcttgaagcctcccaaaggcc<br>aagtgtttccttttct
-150 —<br>-101	ttcttctattttttttcctctctctctaagcgttacttc<br>actgaggca	ttcttctattttttttcctctctctctaagcgttacttc<br>actgaggca

-100 — -51	gagggctgccttggagtgacgtcaggagtttgtg cagcaagcttgcacag	gagggctgccttggagtgacgtcaggagtttgtg cagcaagcttgcacag
-50 — 0	gagaagggaggctgggtgagtgacagcccag cctactttttaatagcttt	gagaagggaggctgggtgagtgacagcccag cctactttttaatagcttt

(Highlight areas are the mutation sequences)
